# 16p11.2 deletion accelerates subpallial maturation and increases variability in human iPSC-derived ventral telencephalic organoids

**DOI:** 10.1242/dev.201227

**Published:** 2023-02-24

**Authors:** Rana Fetit, Michela Ilaria Barbato, Thomas Theil, Thomas Pratt, David J. Price

**Affiliations:** ^1^ Simons Initiative for the Developing Brain, Hugh Robson Building, Edinburgh Medical School Biomedical Sciences, The University of Edinburgh, Edinburgh EH8 9XD, UK; ^2^ Centre for Discovery Brain Sciences, Hugh Robson Building, Edinburgh Medical School Biomedical Sciences, The University of Edinburgh, Edinburgh EH8 9XD, UK

**Keywords:** ASD, 16p11.2 deletion, Ventral telencephalon, Ventral organoids, Ventral progenitors, Cell cycle

## Abstract

Inhibitory interneurons regulate cortical circuit activity, and their dysfunction has been implicated in autism spectrum disorder (ASD). 16p11.2 microdeletions are genetically linked to 1% of ASD cases. However, few studies investigate the effects of this microdeletion on interneuron development. Using ventral telencephalic organoids derived from human induced pluripotent stem cells, we have investigated the effect of this microdeletion on organoid size, progenitor proliferation and organisation into neural rosettes, ganglionic eminence marker expression at early developmental timepoints, and expression of the neuronal marker NEUN at later stages. At early stages, deletion organoids exhibited greater variations in size with concomitant increases in relative neural rosette area and the expression of the ventral telencephalic marker COUPTFII, with increased variability in these properties. Cell cycle analysis revealed an increase in total cell cycle length caused primarily by an elongated G1 phase, the duration of which also varied more than normal. At later stages, deletion organoids increased their NEUN expression. We propose that 16p11.2 microdeletions increase developmental variability and may contribute to ASD aetiology by lengthening the cell cycle of ventral progenitors, promoting premature differentiation into interneurons.

## Teaser

**Highlighted Article:** Three-dimensional region-specific organoids reveal that 16p11.2 deletion increases variability and prolongs the cell cycle of human subpallial progenitors by lengthening their G1 phase.

## INTRODUCTION

Autism spectrum disorder (ASD) is a complex, pervasive neurodevelopmental condition that is characterised by core symptoms which include difficulties in social cognition and communication, repetitive behaviours and hypersensitivities to external stimuli (American Psychiatric Association, 2015; Varghese et al., 2017). The extent of the symptoms varies from patient to patient (London, 2014) and a wide range of comorbidities has been associated with ASD (Canitano, 2007; Hawks and Constantino, 2020; Lai et al., 2019). Evidence suggests that the underlying mechanisms leading to ASD manifestations are a result of early disruptions in the second trimester of foetal development (Wang et al., 2014), the same developmental period when inhibitory cortical interneurons are specified. Therefore, investigating GABAergic interneuron development is particularly relevant to ASD (Marín, 2012; Takano, 2015). Moreover, excitatory/inhibitory imbalance due to interneuron dysfunction has long been considered an important underlying cause of ASD (Rubenstein and Merzenich, 2003; Marín, 2012; Hussman, 2001; Uzunova et al., 2016). Human post-mortem studies provide sufficient evidence to implicate GABAergic and glutamatergic dysfunction in the aetiology of ASD (Fetit et al., 2021).

During development, cortical interneurons arise from the ganglionic eminences (GE) of the ventral telencephalon, which is divided into three proliferative zones, medial, caudal and lateral (MGE, CGE and LGE), distinguished by their expression of different molecular markers. NKX2.1 is highly expressed in the MGE, which generates the largest fraction of the cortical interneurons in both humans and rodents. A smaller proportion of interneurons arise from the CGE, which is marked by abundant COUPTFII (also known as NR2F2) expression. The LGE, on the other hand, makes only a minor contribution to cortical interneuron production (Hansen et al., 2013; Ma et al., 2012; Kelsom and Lu, 2013; Yang et al., 2021). The cortical interneurons produced in the MGE and CGE migrate tangentially through the LGE to integrate with excitatory projection neurons in the cortex (Whalley, 2013).

Although the aetiology of ASD is not yet fully understood, several genetic and environmental factors are known to play a role in its onset and development (De Felice et al., 2015; Varghese et al., 2017). Large genomic copy number variants (CNVs) account for ∼10% of ASD cases (Ramaswami and Geschwind, 2018; Tuzun et al., 2005). 16p11.2 microdeletions, spanning around 600 kb and encompassing 47 genes (Marshall et al., 2008; Pinto et al., 2010), are associated with a variable spectrum of neurocognitive phenotypes. These include ASD, intellectual disability, morbid obesity, macrocephaly or epilepsy at varying degrees of penetrance (Shinawi et al., 2010; Bochukova et al., 2010; Bijlsma et al., 2009; Fetit et al., 2020; Szelest et al., 2021). 16p11.2 microdeletions are also one of the most common genetic linkages to ASD (Fernandez et al., 2010; Weiss et al., 2008).

The underlying molecular mechanisms linking the 16p11.2 deletion to ASD remain largely unknown and have been studied largely in rodent models (Pucilowska et al., 2015; Blumenthal et al., 2014). A number of studies investigating the roles of individual genes within the 16p11.2 locus, such as *MAPK3* (Pucilowska et al., 2015; Mazzucchelli et al., 2002), *QPRT* (Feldblum et al., 1988; Haslinger et al., 2018), *KCTD13* (Golzio et al., 2012) and *TAOK2* (De Anda et al., 2012), suggested possible dysregulation of progenitor proliferation, neuronal migration and cortical lamination (Packer, 2016; Casanova, 2014). Murine models lacking the syntenic region on chromosome 7F3 recapitulate some ASD-like behaviours (Portmann et al., 2014; Pucilowska et al., 2015; Horev et al., 2011; Ouellette et al., 2020; Lu et al., 2019; Angelakos et al., 2017), and exhibit enhanced progenitor proliferation (Pucilowska et al., 2015) and basal ganglia abnormalities (Lu, 2018; Portmann et al., 2014). In addition, evidence from other studies suggests that it is likely that multiple genes within the region interact through shared pathways, contributing to the variable clinical phenotypes (Jensen and Girirajan, 2019; Pizzo et al., 2019).

The advancement of induced-pluripotent stem cell (iPSC) and genome editing technologies has enabled the use of patient-derived tissue to study rare genetic mutations and to model complex neurodevelopmental disorders. Cerebral organoids are 3D-cell aggregates derived from PSCs and contain many of the cell types found in embryonic brains, locally organised and behaving similarly to cells found *in vivo* (Lancaster et al., 2013). To date, only one published study has used iPSC-derived cortical organoids to investigate the effects of the 16p11.2 deletion on cortical development (Urresti et al., 2021). In addition to recapitulating the patient macrocephalic phenotype, 16p11.2 patient-derived cortical organoids exhibited an excess of neurons and depletion of neural progenitors (Urresti et al., 2021).

To date, there are no reports of using region-specific ventral organoids to specifically address the effects of this deletion on interneuron development. Here, we demonstrate that ventral organoids harbouring the deletion are more variable in size than normal. They exhibit significant relative increases in neural rosette area, COUPTFII expression at earlier timepoints and prolonged cell cycle length primarily due to lengthening G1. All these properties are significantly more variable in deletion organoids. Additionally, deletion organoids exhibit increased NEUN (also known as RBFOX3) and LHX6 mean fluorescence intensity at later timepoints. Our results suggest increased variability and accelerated maturation of ventral deletion organoids, which may result in the premature differentiation of ventral progenitors into interneurons.

## RESULTS

### Off-target genetic variation between cell lines was limited

We adapted a protocol described previously (Sloan et al., 2018) to generate ventral telencephalic organoids from heterozygous 16p11.2 CRISPR/Cas9-deletion and isogenic control iPSC lines, all derived from the same parent line, GM08330, which is referred to as GM8 in this study (Tai et al., 2016). No significant off-target CNVs were observed in previous studies of these CRISPR/Cas9-generated clones (Tai et al., 2016; Sundberg et al., 2021; Lim et al., 2022).

We used Illumina CytoSNP array analysis to confirm the presence of the 16p11.2 heterozygous deletion in all three deletion iPSC lines used in this study (Fig. S1). The parent line, GM8, and the three isogenic control lines did not carry any deletions or duplications of the 16p11.2 region. Our analysis also revealed a number of genomic locations where loss of heterozygosity (LOH) without a loss in copy number (copy neutral LOH) had occurred, usually in all lines (green bands, Fig. S1; Table S2). Of the few additional deletions and duplications that were present in some lines and not others (red, yellow, blue and purple bands, Fig. S1; Table S2), only five of the affected regions contained protein-coding genes, as follows. (1) A heterozygous deletion involved one protein-coding gene on chromosome 4 in control lines FACS52 and FACS53. (2) A heterozygous duplication involved six protein coding genes on chromosome 4 in deletion line DELD5. (3) The control line FACS53 had three additional CNVs: a heterozygous deletion on chromosome 5 involving one gene, a heterozygous duplication involving three genes on chromosome 4 and a heterozygous duplication involving eight genes on chromosome 9. We designed our experiments to take into account the possibility that the effect of 16p11.2 deletion might be modified by these few heterozygous variants.

**Fig. 1.**
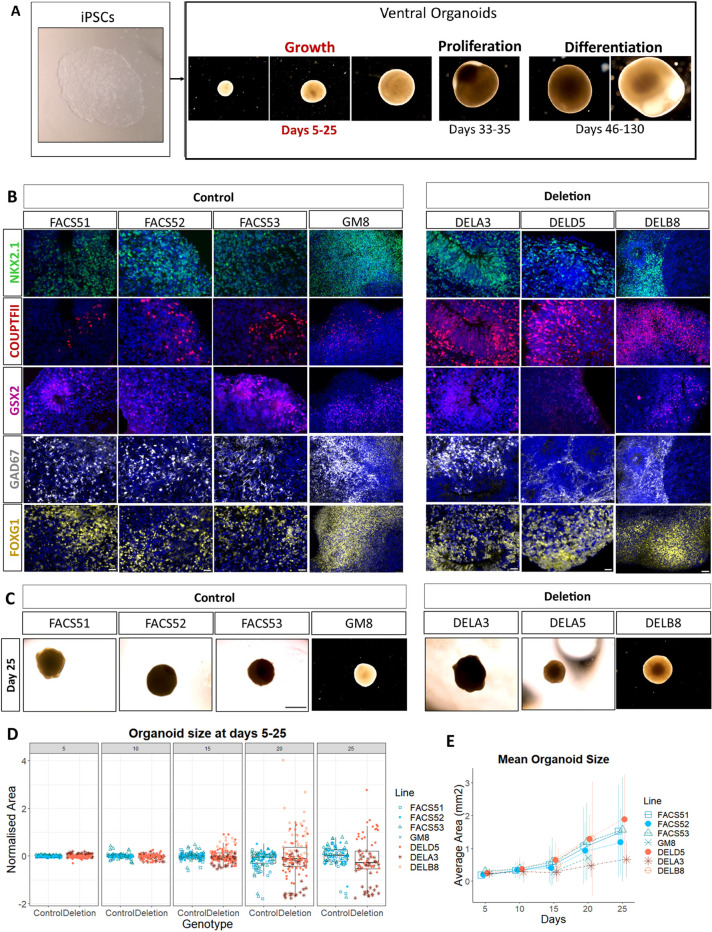
**Ventral organoids express ventral telencephalic markers and exhibit variations in size.** (A) Overview of the study design with representative images of organoids. The first part focused on organoid growth by measuring organoid size between 5 and 25 days. The second part focused on the proliferation of ventral progenitors by analysing cell cycle kinetics at 33-35 days. The third part examined their differentiation through the expression of neuronal markers at 46-130 days. (B) Both control and deletion organoids express the GE markers NKX2.1, COUPTFII and GSX2, as well as the forebrain marker FOXG1 and the GABAergic marker GAD67 at days 33-35. Images are shown merged with DAPI (blue). Scale bars: 25 µm for the cell lines FACS51, FACS52, FACS53, DELA3 and DELD5; 50 µm for the cell lines GM8 and DELB8. (C) Representative images of the organoids at day 25. Scale bar: 1 mm. (D) Projected area of ventral organoids over a period of 25 days. Box plots showing organoid area (mm^2^) normalised to the average of the control area for every batch at different timepoints. Data are pooled from the different replicates across four different batches. Every data point represents the result from a single organoid. Data for the lines GM8 and DELB8 used in batch RF5 for part 3 of the study are also shown at days 15 and 20. Different shapes represent the different cell lines used. Boxes represent the interquartile range with median; whiskers represent the highest and lowest values. (E) Average organoid area (absolute values) for every cell line at the different timepoints. Data are mean±2×s.d.). Sample size by genotype: day 5, control=152, deletion=149; day 10, control=137, deletion=131; day 15, control=132, deletion=147; day 20, control=100, deletion=130; day 25, control=85, deletion=74.

This study was divided into three parts. The first part investigated initial organoid growth between 5 and 25 days. The second part focused on the cell cycle kinetics of ventral progenitors at days 33-35 and the third part assessed the differentiation of daughter cells between days 46-130 (Fig. 1A, Table S1). Organoids from the four control lines (FACS51, FACS52, FACS53 and GM8) and three deletion lines (DELD5, DELA3 and DELB8) were used to assess organoid growth over a period of 25 days. To assess proliferation, organoids were grown for 33-35 days from three control lines (FACS51, FACS52 and FACS53) and two deletion lines (DELD5 and DELA3). Each of four separate batches of organoids (RF1-RF4) included at least one control and one deletion line. Across the four batches, all lines were replicated twice, except FACS53, which was replicated three times, and DELD5, which was replicated four times. All replicates included 10-15 organoids. To assess differentiation at later stages, a batch (RF5) of ventral organoids from two of these lines (GM8 and DELB8) containing 4-10 organoids per line was maintained for 46-130 days (Table S1). A combination of linear mixed effects (LME) analysis, Welch's ANOVA and Student's *t*-test was used to account for the variability due to both batch-effects and cell line differences, and assess the statistical significance of our findings.

### Deletion and control organoids developed ventral telencephalic identity

We first characterised the organoids by immunohistochemistry (IHC) to assess their expression of forebrain and ventral telencephalic markers. At days 33-35, ventral organoids from the control and deletion lines used in parts 1 and 2 to assess growth and proliferation, as well as the lines used in part 3 of the study to assess differentiation expressed the GE markers NKX2.1, COUPTFII and GSX2, together with the forebrain marker FOXG1 and the GABAergic marker GAD67 (Fig. 1B). In addition, the ventral organoids did not express the dorsal telencephalic markers EMX1 and TBR2 (Fig. S2A,B). These findings confirmed that the protocol used here indeed generated control and deletion organoids of ventral telencephalic identity.

### Early 16p11.2 deletion organoids exhibited abnormal variations in size

We assessed organoid growth because macrocephaly is frequently reported in individuals with 16p11.2 deletions (Qureshi et al., 2014) and increases in the volume of subcortical GE-derived brain structures, such as the striatum and globus pallidus, have been shown in 16p11.2 deletion mice (Rein and Yan, 2020). The projected area of ventral organoids from both deletion and control lines was measured every 5 days over a period of 25 days. Fig. 1C shows representative organoids from the different lines at day 25. Fig. 1D shows data on individual organoid areas normalised to the average area of the control organoids in the same batch at the same age (Table S3). The mean organoid areas for each line are shown in Fig. 1E. The organoids from the control lines all grew at relatively similar rates compared with the organoids from the deletion lines, the sizes of which became more variable from day 15 on (*F*-test for comparing variance between genotypes, *P*=2.30e-07, 4.12e-07 and 5.23e-10 for days 15, 20 and 25, respectively). This variation was between batches of deletion organoids rather than between deletion lines (Fig. S3A). Taking batch variability into account, LME analysis revealed no significant effects of the genotype on organoid area at day 25 (*P*=0.113, type III ANOVA). Our findings showed that 16p11.2 ventral organoids were not consistently larger or smaller but exhibited significantly greater variation in growth rates compared with their batch-matched controls.

### 16p11.2 deletion organoids exhibited increased potential for neural rosette formation

**Fig. 2.**
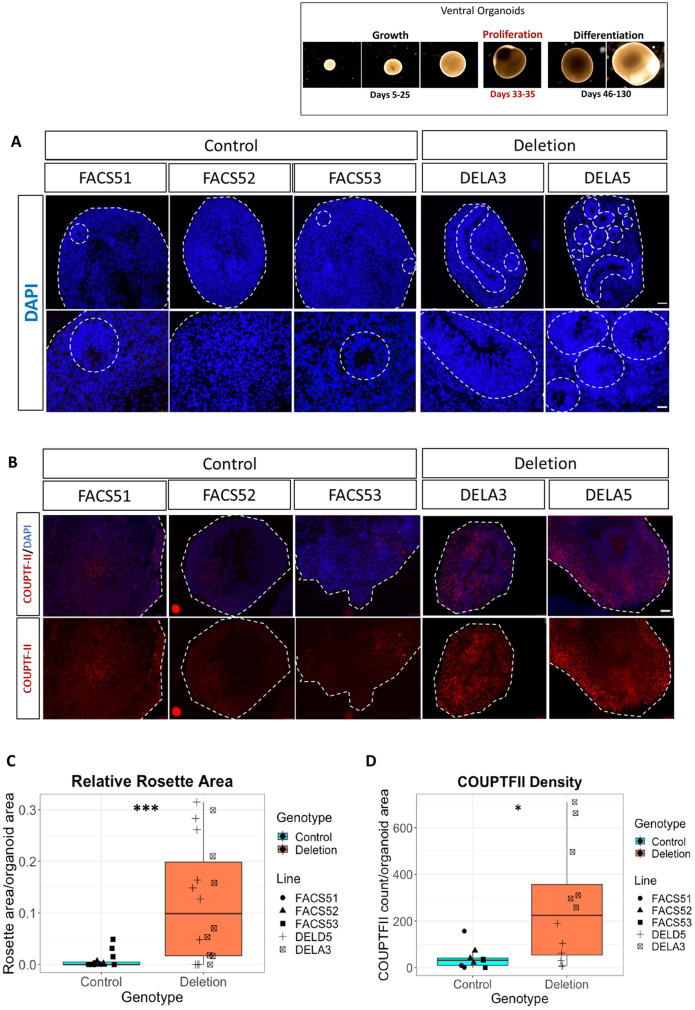
**Quantification of neural rosettes and COUPTFII expression in ventral organoids at day 35.** (A) Rosettes in representative ventral organoids from control and deletion lines. Scale bars: 25 µm (bottom) and 100 µm (top). Organoid perimeter and rosettes are outlined by dashed lines. (B) COUPTFII expression in control and deletion lines. Top panels show COUPTFII and DAPI, bottom panels show the red channel for COUPTFII only. Scale bar: 100 µm. (C) Box plots showing the rosette area relative to organoid area grouped by genotype (control *n*=15, deletion *n*=18). Statistical significance was determined using Welch's ANOVA (***P=0.000564). (D) Box plots showing COUPTFII density (COUPTFII counts in the whole-organoid section/organoid size). Data are grouped by genotype (control *n*=9, deletion=12). Statistical significance was determined using Welch's ANOVA (*P=0.01093). Every data point represents the result from a single organoid. Boxes represent the interquartile range with median; whiskers represent the highest and lowest values.

Given that several genes within the 16p11.2 deletion region are expressed in neural progenitor cells (NPCs) (Morson et al., 2021), we investigated whether the deletion affects the size and abundance of neural rosettes, which are radial arrangements of NPCs that resemble the developing neuroepithelium (Deglincerti et al., 2016; Townshend et al., 2020). We delineated the rosette structure based on the radial circular arrangement of NPCs using DAPI (Fig. 2A). Neural rosettes often appeared larger and more abundant in organoids from deletion lines compared with controls (Fig. 2A). This was observed in all three deletion lines at days 33-35 (Fig. 2A and Fig. S2D). We quantified the area occupied by the neural rosettes relative to the area of the organoid from the batches RF1-4 (Table S5, Fig. S3B), thereby taking variations in organoid size into account. Compared with controls, significantly larger average relative rosette area was found in deletion organoids (Welch's ANOVA, *P*=0.000564; large effect size, Cliff's delta=0.6518519; Fig. 2C). Post-hoc comparisons of the individual lines are summarised in Table S4. Deletion organoids exhibited significantly greater variation in relative rosette area compared with their isogenic controls, with some deletion organoids in some batches generating very few rosettes (*F*-test, *P*=5.382e-10; Fig. S3B). There was no obvious relationship between whether deletion organoids increased their relative rosette area and their size (Fig. S3C, Spearman correlation R=−0.12, *P*=0.71). In addition, we observed no remarkable differences in rosette morphology and arrangement of NPCs around the inner lumen between deletion and control organoids (Fig. S2C). Collectively, our findings suggest that 16p11.2 deletion increased the potential of ventral organoids to form neural rosettes and increased the variation in rosette generation.

### 16p11.2 deletion organoids exhibited a significant increase in COUPTFII-expressing cell density

While characterising ventral organoids as described above, we noticed that COUPTFII was expressed by many more cells in organoids from both deletion lines than in controls (Fig. 2B). Given that the cell density might vary across an organoid, we consistently assessed the expression of COUPTFII in sections from the middle of the organoid. The number of cells expressing COUPTFII in whole-organoid sections was then quantified and COUPTFII^+^ areal cell density was calculated, thereby taking into account the variations in organoid size. A significant increase in COUPTFII^+^ cell density was observed in the deletion organoids compared with their isogenic controls (Welch's ANOVA, *P*=0.01093; large effect size, Cliff's delta=0.5925926, Fig. 2D). Post-hoc pairwise comparisons of the individual lines are summarised in Table S4. Deletion organoids exhibited significantly greater variation in COUPTFII^+^ cell density, with some deletion organoids in some batches generating relatively few COUPTFII^+^ cells (*F*-test, *P*=9.968e-05; Fig. S3D). There was no relationship between whether deletion organoids increased their relative rosette area and their density of COUPTFII^+^ cells (Fig. S3E). Organoids from the deletion line (DELB8) used in the third part of this study also recapitulated this increased COUPTFII expression at the same timepoints, but this was not quantified (Fig. S2E). Quantification of the relative mean expression of NKX2.1, GSX2 and FOXG1 at day 35 revealed no significant difference between control and deletion lines (Fig. S4A-D, Table S10, Welch two-sample *t*-test, *P*=0.8427, 0.1059 and 0.6645 for the three markers, respectively). These findings suggest that ventral organoids harbouring the 16p11.2 deletion have the potential to generate proportionately more COUPTFII^+^ cells and exhibit increased variability in COUPTFII^+^ cell density.

### 16p11.2 deletion did not alter the proportions of proliferating progenitors at early timepoints

**Fig. 3.**
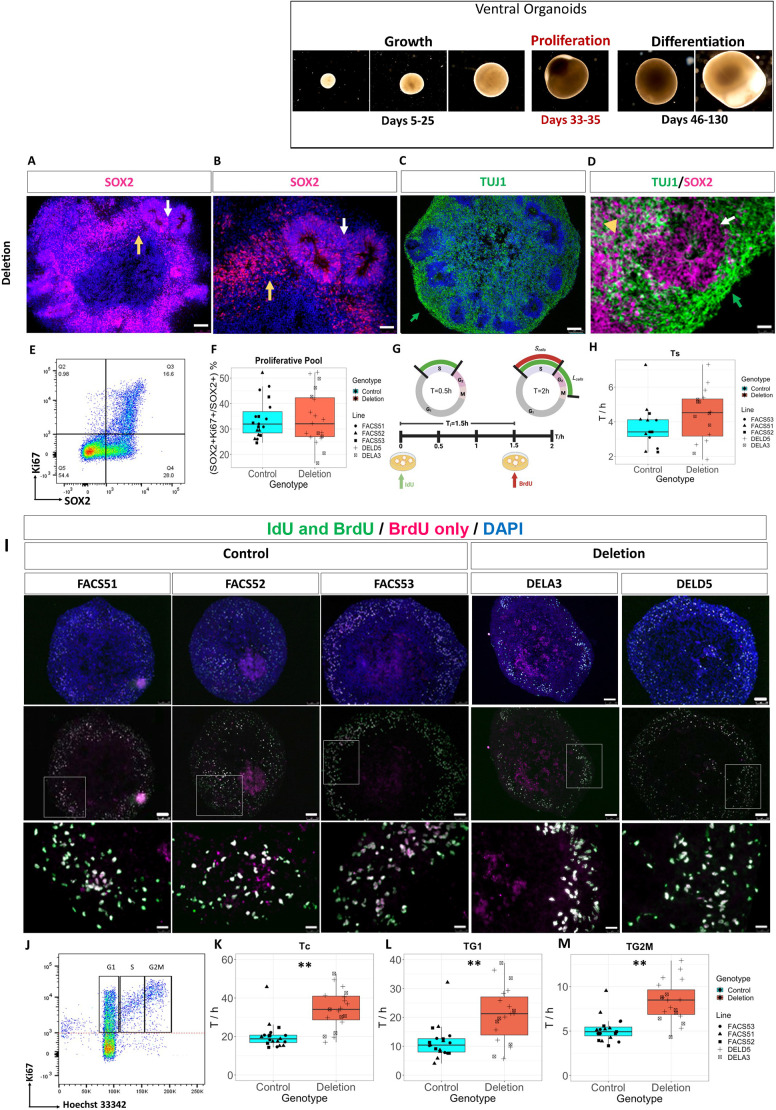
**Cell cycle kinetics in ventral organoids.** (A,B) SOX2^+^ cells (magenta) reside in the outer rim of the ventral organoids (yellow arrows) and in neural rosettes (white arrows). Scale bars: 100 µm in A; 50 µm in B. A representative rosette from a deletion organoid where rosettes were more abundant is shown. (C) TUJ1^+^ late NPCs and immature neurons (green) reside outside neural rosettes in the organoid periphery (green arrow). (D) A neural rosette positive for SOX2 (magenta, white arrow) and surrounded by TUJ1^+^ immature neurons (green arrow). Some TUJ1^+^ cells also express SOX2 (yellow arrowhead). Scale bar: 25 µm in C and D. (E) Flow cytometric density plot showing SOX2 (*x*-axis) against Ki67 (*y*-axis) in a representative organoid. In this organoid, 37% of the SOX2^+^ cells are cycling and positive for the cell cycle marker Ki67. (F) The proportion of SOX2^+^Ki67^+^ progenitors as percentages of all SOX2^+^ cells in the organoid (proliferative pool). Sample size by genotype: *n*=21 organoids for control and deletion. Sample size by cell line: FACS51, *n*=9; FACS52, *n*=5; FACS53, *n*=7; DELD5, *n*=14; DELA3, *n*=7. (G) Schematic representation of the IdU/BrdU double-labelling experiment (created using BioRender.com). (H) Duration of S phase (Ts) in hours (h) calculated from double IdU/BrdU labelling. (I) Representative images of ventral organoids stained with monoclonal antibodies specific for both IdU and BrdU (green) and BrdU only (magenta) to allow for the identification of S_cells_ and L_cells_. Top panels show organoids at 10× magnification merged with DAPI (blue). Middle panels show the green and magenta channels. Scale bars: 100 µm. White boxes outline the regions magnified in the lower panels. Scale bars: 25 µm. (J) Density plot of Ki67 (*y*-axis) against Hoechst 33342 (*x*-axis). SOX2^+^ cells in the cell cycle are those positive for Ki67 (above the dotted red line). Cells in G_1_ are those with 2n DNA content and those in G_2_M are those with double the DNA content (4n). In between are cells in S phase. (K-M) Duration of the total cell cycle (T_c_) and of the individual cell cycle phases in ventral organoids grouped by genotype in hours (h). Statistical significance determined using LME analysis to account for batch variability. **P=0.001733 for T_c_, 0.008776 for T_G1_ and 0.001826 for T_G2M_ (type III ANOVA). Sample size by genotype: control, *n*=19; deletion, *n*=19. Sample size by cell line: FACS51, *n*=8; FACS52, *n*=5; FACS53, *n*=6; DELD5, *n*=12; DELA3, *n*=7. Every data point represents the result from a single organoid analysed by flow cytometry. Boxes represent the interquartile range with median; whiskers represent the highest and lowest values.

Neural rosettes can respond to different patterning cues and initiate differentiation into region-specific neuronal fates (Elkabetz et al., 2008). Because more developed neural rosettes were observed in the deletion organoids, we then asked whether the deletion affected the proportions of proliferating progenitors in the organoids. We observed that SOX2, a marker associated with early stage NPCs and relatively undifferentiated precursor cells (Pagin et al., 2021), was expressed in neural rosettes (white arrows, Fig. 3A,B,D) and cells surrounding them in the outer region of the ventral organoids (yellow arrows, Fig. 3A,B); TUJ1 (green arrows, Fig. 3C,D), a marker expressed in late-stage neurogenic NPCs and immature newly generated postmitotic neurons (von Bohlen und Halbach, 2007; Liu et al., 2007), was expressed by cells surrounding the rosettes in the outer regions of the organoids (yellow arrowhead, Fig. 3C,D). This was observed in both deletion and control organoids, although the control organoids did not form many rosettes.

To determine the proportions of cycling early proliferative (SOX2+Ki67+/SOX2+) progenitors and late neurogenic (TUJ1+Ki67+/SOX2+) progenitors, days 33-35 organoids were dissociated, fixed and permeabilised, then analysed with flow cytometry (Fig. S8). Cells were labelled for TUJ1, SOX2 and the cell cycle marker Ki67 to unambiguously identify the cycling progenitor cells, which we will refer to as the proliferative pool (Fig. S7). Not all SOX2^+^ cells expressed Ki67 (Fig. 3E), indicating the SOX2 expression persists after cells exit the cell cycle, which was also observed for day 30 ventral organoids in a recently published study (Xiang et al., 2017) (Fig. S5A).

We first quantified the proportion of the SOX2^+^ cells that were proliferative (SOX2+Ki67+cells/SOX2+ cells) in our ventral organoids (Fig. 3F, Fig. S5B). We found no significant differences between deletion and control organoids in either average proportions (taking batch variability into account with LME analysis; *P*=0.9748, type III ANOVA) or variability (*P*=0.1977, *F*-test; Fig. S5B). We then quantified the proportion of proliferative TUJ1^+^ late NPCs to assess the proportion of NPCs primed towards neurogenesis (TUJ1+Ki67+cells/SOX2+ cells) and found no significant differences between deletion and control organoids (Fig. S5C,D, Table S9, LME analysis; *P*=0.4891, type III ANOVA). Moreover, deletion organoids displayed a slight increase in the ratio of proliferating late (TUJ1+Ki67+) NPCs to early (SOX2+Ki67+) progenitors, albeit statistically insignificant (Fig. S5E,F; LME analysis; *P*=0.2079, type III ANOVA). This was accompanied with significant variability in the ratio of late-neurogenic:early-proliferative NPCs observed in the deletion organoids compared with the controls (*F*-test, *P*=0.03928). Our findings suggest that, at this developmental timepoint (day 33-35), 16p11.2 microdeletion increases the potential of ventral progenitors to organise into neural rosettes without affecting the proportions of either early proliferative or late neurogenic progenitors in the deletion organoids.

### 16p11.2 deletion organoids exhibited increased total cell cycle lengths and lengthened G1 and G2M phases

Studies in 16p11.2 mouse models and patient-derived NPCs have demonstrated enhanced cortical progenitor proliferation (Pucilowska et al., 2015; Connacher et al., 2022), whereas other studies using human iPSC-derived cortical NPCs showed no difference in NPC proliferation rates (Deshpande et al., 2017; Roth et al., 2020). Therefore, we investigated whether the deletion affects the cell cycle of ventral progenitors within the outer SOX2^+^ peripheral region of the organoids, as control organoids formed very few rosettes. First, we determined the length of S phase (Ts) using double iododeoxyuridine (IdU) and bromodeoxyuridine (BrdU) labelling, as previously described (Martynoga et al., 2005) (Fig. 3G,I, Table S5). Although deletion organoids revealed a slight increase in the duration of S phase (Ts) (Fig. 3H), this was not statistically significant (LME analysis; *P*=0.1017, type III ANOVA). There was no significant difference in variation between control and deletion organoids (Fig. S6A; *F*-test, *P*=0.517).

The numbers of SOX2^+^ cells that were proliferative, as marked by Ki67 expression, were calculated from flow cytometry (Fig. 3E), together with the number of these cells in the different cell cycle phases (Fig. 3J; Table S6). These data were used to calculate the total cell cycle length (Tc) and the duration of G1 (T_G1_) and G2M (T_G2M_) phases, as described previously (Martynoga et al., 2005). The average total cell cycle length (T_C_) for the deletion organoids was 33.8 h, which was significantly higher than that of the control organoids (20.1 h) (Table S6; LME analysis; *P*=0.001733, type III ANOVA; large effect size, omega_squared=0.40, Fig. 3K, Fig. S6B). Moreover, significant increases in the duration of G1 (T_G1_) and G2M (T_G2M_) phases were observed in the deletion organoids compared with controls (Fig. 3L,M, LME analysis; *P*=0.008776 and 0.001826 for T_G1_ and T_G2M_, respectively, type III ANOVA). The effect sizes were large in both cases (Omega_squared=0.37 and 0.83 for T_G1_ and T_G2M_, respectively). In addition, deletion organoids exhibited greater variations in T_G1_ and T_G2M_ compared with controls (Fig. S6C,D; *F*-test, *P*=0.05052 and 0.04201 for T_G1_ and T_G2M_, respectively). These findings suggest an elongation of the cell cycle due to increased G1 and G2M phase length in deletion organoids, concomitant with increased variability.

### Longer cell cycles correlated with lengthened G1 phase and increased relative rosette area in deletion organoids

**Fig. 4.**
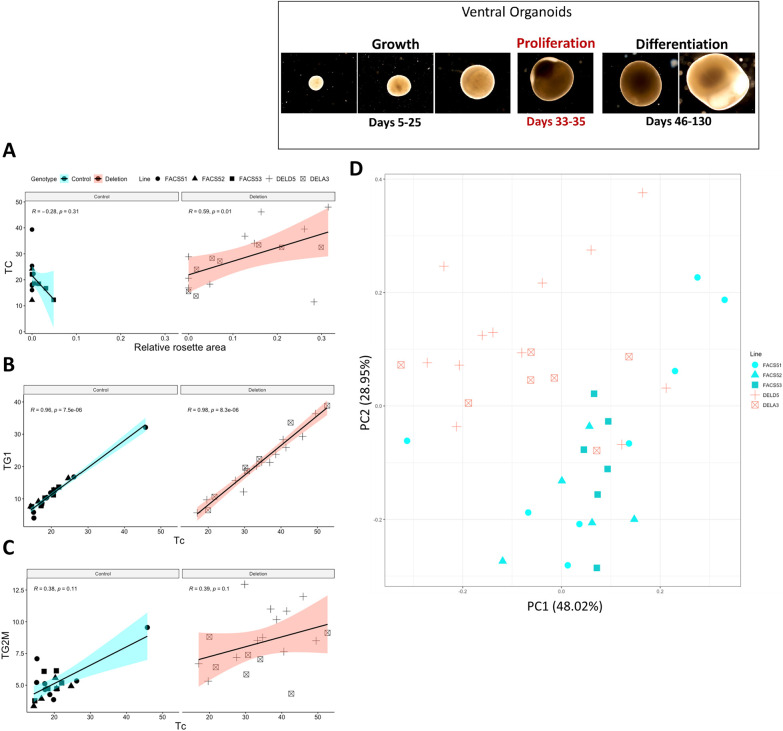
**Linking relative rosette area, T_G1_ and T_G2M_ to T_C_ in imaging and flow cytometry datasets.** (A) Correlation analysis between T_C_ and relative rosette area in the imaging dataset (Spearman correlation). (B,C) Correlation analysis between T_C_ and T_G1_ (B) and T_G2M_ (C) in the flow cytometric dataset (Spearman correlation). Control, *n*=19; deletion, *n*=19. (D) PCA analysis of the organoids in flow cytometric dataset, grouped by cell line. Every data point represents the result from a single organoid. Different shapes correspond to the different cell lines used.

We then asked whether the increased relative rosette area is correlated with longer Tc. Therefore, we calculated Tc for the organoids analysed in the imaging dataset and performed a correlation analysis between relative rosette area and Tc (Table S5, Fig. 4A). Organoids from the two deletion lines behaved similarly, in that those exhibiting high relative rosette area also exhibited longer Tc. Correlation analysis revealed a significantly stronger positive correlation between relative rosette area and Tc in deletion organoids than in controls, where a negative but insignificant correlation was observed (Fig. 4A, Spearman correlation, R=0.59 *P*=0.01 and R=−0.28 *P*=0.31 for deletion and controls, respectively). There was no evidence of a correlation between COUPTFII^+^ cell density and Tc (Fig. S6E).

After that, we examined the relationship between Tc, T_G1_ and T_G2M_ in the organoids analysed by flow cytometry (Table S6). We can clearly see that T_G1_ increases with longer Tc, a correlation that was both strong and significant in the deletion and control organoids (Fig. 4B, Spearman correlation, R=0.98 *P*=8.3e-06 and R=0.96 *P*=7.5e-06 for deletion and control organoids, respectively). The correlation between T_G2M_ and Tc was rather moderate and insignificant (Fig. 4C; Spearman correlation, R=0.38, *P*=0.1 and R=0.39, *P*=0.1 for control and deletion, respectively).

We then ran a principal component analysis (PCA) using all variables generated in the flow cytometric dataset to observe how similar or different the individual organoids are within the control and deletion populations. PCA clearly separates most deletion organoids from the controls (Fig. 4D). Taken together, we can conclude that the increase in relative rosette area in the deletion organoids correlates with longer Tc, which is primarily due to the lengthening of G1 phase. Moreover, the effects of the 16p11.2 CNV are similar in organoids from both deletion lines. Tables S5 and S6 list all the variables measured and calculated for every organoid in the imaging and flow cytometric datasets.

### 16p11.2 deletion organoids exhibited increased levels of TUJ1 and GAD67 at early time points

**Fig. 5.**
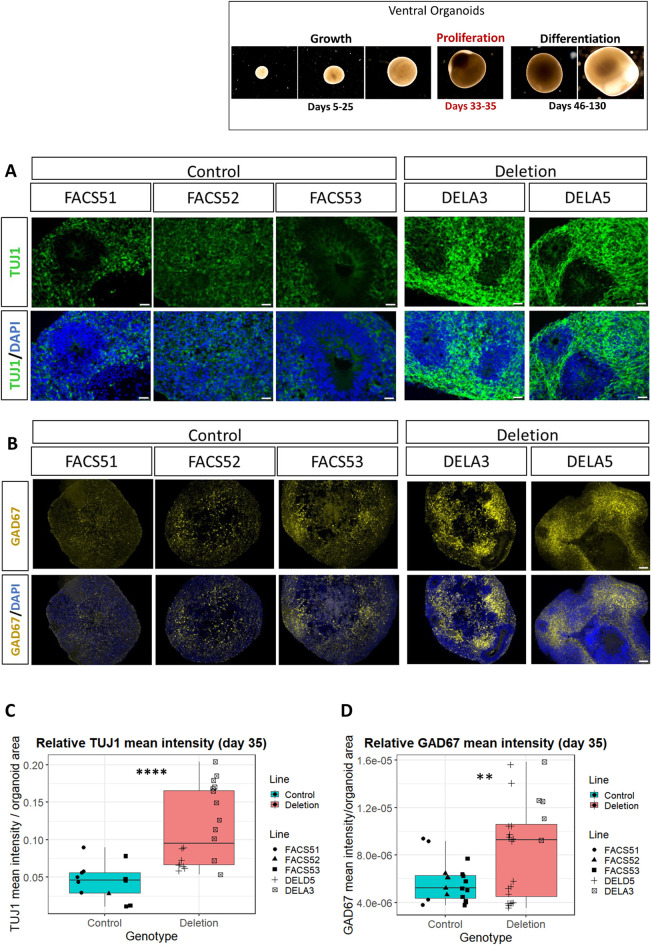
**Relative TUJ1 and GAD67 mean fluorescence intensity in ventral organoids at 35 days.** (A) TUJ1 expression in control and deletion lines. Top panels show TUJ1 only, bottom panels show TUJ1 and DAPI. Scale bars: 25 µm. (B) GAD67 expression in representative whole-organoid sections from the control and deletion lines. Top panels show GAD67 only, bottom panels show GAD67 and DAPI. Scale bars: 100 µm. (C,D) Mean TUJ1 and GAD67 fluorescence intensity relative to whole-organoid area. ****P=2.161e-05 (C) and **P=0.003701 (D) (Welch's two sample *t*-test). Every data point represents the result from a single organoid. Boxes represent the interquartile range with median; whiskers represent the highest and lowest values.

Because cell cycle and G1-phase lengthening are features of neural stem cells undergoing neurogenic cell divisions (Wilcock et al., 2007; Molina et al., 2020 preprint), we investigated whether this may affect progenitor differentiation in deletion organoids. The mean fluorescence intensity of TUJ1 and the GABAergic interneuron marker GAD67 were quantified relative to organoid size at days 33-35 (Fig. 5A,B, Table S11). A significant increase in the relative mean fluorescence intensity of both markers was observed in the deletion organoids, which exbibited increased variability compared with controls (Fig. 5C,D; *P*=2.161e-05 and 0.003701, Welch's two-sample *t*-test; *P*=0.01338 and 0.0004275, *F*-test, for TUJ1 and GAD67, respectively). The increased TUJ1 and GAD67 expression observed in deletion organoids, concomitant with increased variability, suggests that the differentiation into interneurons might begin earlier in ventral organoids with 16p11.2 deletion.

### 16p11.2 deletion organoids exhibited increased *NEUN* and *LHX6*, followed by significant reductions in LHX6 at later stages

**Fig. 6.**
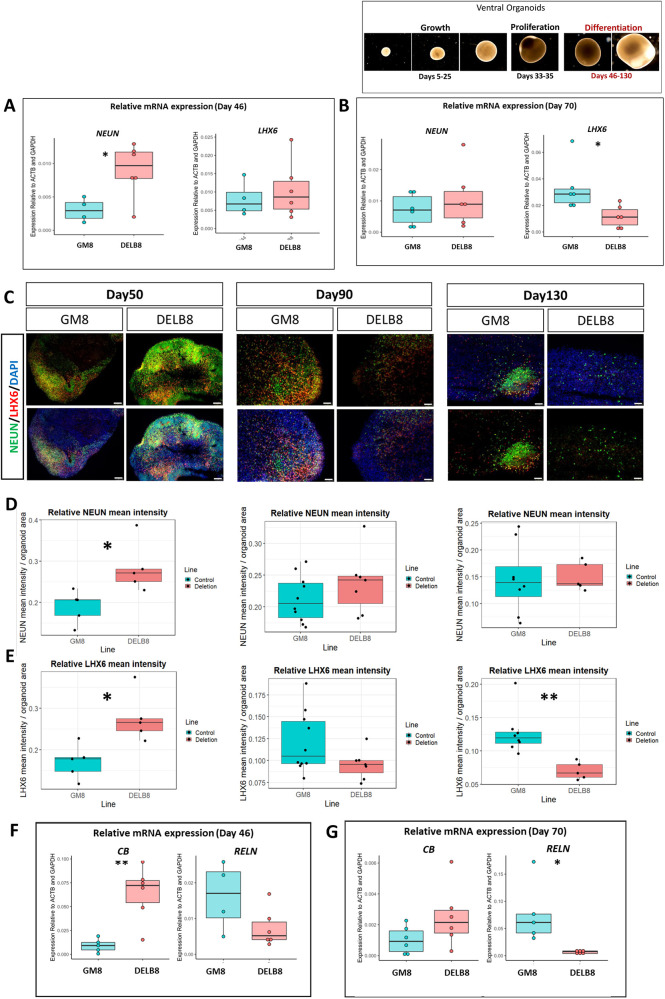
**RT-qPCR and immunohistochemical analysis of interneuron markers and transcription factors in ventral organoids from the cell lines GM8 and DELB8.** (A,B) Box plots showing relative mRNA expression of NEUN and LHX6 in control (blue) and deletion (red) organoids at 46 and 70 days of differentiation, respectively. *n*=4-6 organoids, *P<0.05 (unpaired Student's *t*-test). (C) NEUN and LHX6 expression at days 50, 90 and 130 in representative control and deletion organoids. Top panels are merged with DAPI; bottom panels show cells expressing NEUN and LHX6 only. Scale bars: 100 µm. (D,E) Mean fluorescence intensity of NEUN and LHX6 relative to organoid size quantified from whole-organoid immunohistochemical sections at days 50, 90 and 130. *P*=0.02311 for NEUN at day 50, *P*=0.01229 and 0.00133 for LHX6 at days 50 and 130, respectively (Welch two sample *t*-test). (F,G) Relative mRNA expression of calbindin (CALB1) and reelin (RELN) at 46 and 70 days. *n*=4-6 organoids, *P<0.05, **P<0.005 (unpaired Student's *t*-test). Every data point represents the result from a single organoid. Boxes represent the interquartile range with median; whiskers represent the highest and lowest values.

To investigate whether precocious development in ventral organoids with 16p11.2 deletion results in premature differentiation into interneurons, we generated an additional batch of ventral organoids from the parent line (GM8) and the deletion line (DELB8) containing four to six organoids per line. Organoids were maintained for 130 days and the expression of NEUN, a marker of neuronal maturation that labels mature neurons (Gusel'Nikova and Korzhevskiy, 2015), and LHX6, a transcription factor that regulates MGE interneuron production (Marín, 2012) and is expressed in MGE-derived interneurons (Voss et al., 2022), was examined. A significant increase in *NEUN* mRNA expression was observed at day 46 in deletion organoids compared with their isogenic controls (Fig. 6A; *P*<0.05, unpaired Student's *t*-test), with no difference in *LHX6* mRNA expression (Fig. 6A). In contrast, no differences in *NEUN* mRNA expression were found at day 70, yet a significant reduction in *LHX6* mRNA expression was observed in the deletion organoids (Fig. 6B; *P*<0.05, unpaired Student's *t*-test).

We then examined NEUN and LHX6 expression at days 50, 90 and 130 by immunohistochemistry (Fig. 6C). At day 50, a significant increase in the mean NEUN and LHX6 fluorescence intensity relative to organoid size was observed in the deletion organoids (Fig. 6D,E, Table S7; *P*=0.02311 and 0.01229, Welch's two-sample *t*-test, for NEUN and LHX6, respectively). At day 90, no significant differences in the mean NEUN and LHX6 fluorescence intensity relative to organoid size were observed (Fig. 6D, E; *P*=0.2856 and 0.06599,Welch's two-sample *t*-test, for NEUN and LHX6, respectively). By day 130, no significant differences were observed in NEUN mean fluorescence intensity at day 130 (Fig. 6D; *P*=0.8426, Welch's two-sample *t*-test); however, the deletion organoids revealed a significant reduction in LHX6 mean fluorescence intensity (Fig. 6E; *P*=0.00133, Welch's two-sample *t*-test). In addition, we observed a significant reduction in NEUN expression between days 90 and 130 for both control and deletion organoids (Table S7; *P*=0.0029 and *P*=0.0238814, unpaired Student's *t*-test, in the deletion and control organoids respectively). Taken together, our findings suggest that the 16p11.2 deletion accelerates neuronal output in ventral organoids, which leads eventually to the production of fewer interneurons at later stages.

### 16p11.2 deletion organoids exhibited increased calbindin expression

Many mature MGE-derived cortical interneurons express calbindin 1 (CALB1). Similarly, mature interneurons expressing reelin (RELN) are CGE derived. Higher levels of CALB1 mRNA expression were found in deletion organoids, which was significant at day 46 but not day 70 (Fig. 6E,F; *P*<0.05, unpaired Student's *t*-test). Lower *RELN* levels were found in the deletion organoids at the two timepoints and this was significant at day 70 (Fig. 6E,F; *P*<0.05, unpaired Student's *t*-test). No significant differences in somatostatin (*SST*), calretinin (*CALB2*) and neuropeptide Y (*NPY*) expression were seen (Fig. S7B), although there was a trend towards a slight increase in their relative expression in the deletion organoids at day 70. Taken together, our findings suggest that this CNV affects interneuron differentiation and may lead to the preferential formation of *CALB1* interneurons at the expense of *RELN* interneurons.

## DISCUSSION

### Summary of the effects of 16p11.2 deletion in ventral organoids

**Fig. 7.**
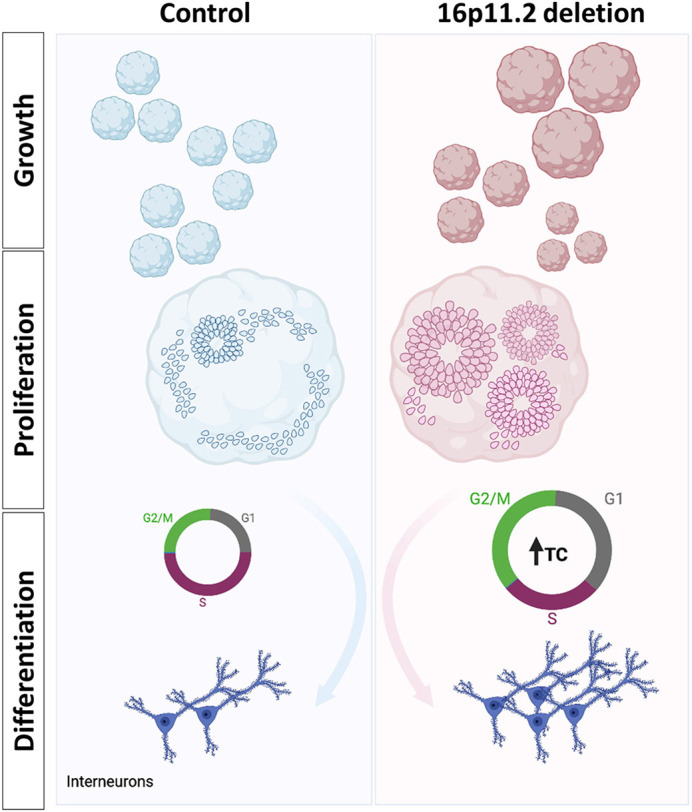
**Summary of the effects of 16p11.2 deletion in ventral organoids.** Control organoids from the different lines grew at similar rates, whereas deletion organoids exhibited significant variations in growth rates. Regardless of organoid size, deletion organoids from both lines displayed a greater potential to form rosettes without altering the numbers of cycling progenitors at early timepoints, with increased variability in the observed phenotype. However, cycling progenitors in the deletion organoids proliferated more slowly than the controls. The effect of this prolonged cell cycle length, as well as the duration of G1 and G2M, becomes evident at later timepoints, where the expression of the neuronal markers NEUN and LHX6 is increased, suggesting that this CNV accelerates neuronal production and has the potential to alter interneuron subtypes. Created using BioRender.com.

To our knowledge, this is the first study to use ventral telencephalic organoids to investigate the effects of 16p11.2 deletion on early aspects of interneuron development in the ventral telencephalon. The first signs of an effect came from the increased variability in the growth of deletion organoids over the first few weeks of their development, although their average growth rates did not differ significantly from those of controls. Subsequently, at stages when telencephalic organoids start to organise their neural cells into rosettes in a process that resembles the development of the neuroepithelium of the neural tube, deletion organoids showed, on average, increased rosette formation. This increased state of organisation coincided with higher densities of COUPTFII-expressing cells and longer cell cycles (Wilcock et al., 2007; Molina et al., 2020 preprint). As with the growth rates recorded at earlier stages, the relative rosette area, COUPTFII density, and the duration of G1 and G2M phases varied more in deletion organoids than in controls. Our study also suggested that, at earlier stages, the deletion organoids start forming interneurons precociously, thereby resulting in lower neuron numbers at later stages and may potentially alter the subtypes of interneurons produced. These findings are summarised in Fig. 7.

### Variability as a hallmark of 16p11.2 deletion

In this study, deletion organoids consistently exhibited increased variability compared with their isogenic control organoids. This was evident by the increased variability observed in the rosette area, COUPTFII expression, cell cycle kinetics as well as TUJ1 and GAD67 expression among deletion organoids. The very similar batch-batch variation observed in both deletion lines (Figs S2 and S3) argued against a significant contribution from the heterozygous duplication affecting six protein-coding genes on chromosome 4 in deletion line DELD5 and any other genetic differences between these effectively isogenic lines.

This variability is important when considering why individuals with 16p11.2 deletion also demonstrate a variable clinical phenotype. The clinical heterogeneity and incomplete penetrance in such individuals are indeed very remarkable, thus rendering the correct clinical diagnosis challenging (Fetit et al., 2020). We propose that the loss of this region, which contains several genes that are involved in the regulation of the cell cycle during neurogenesis and that converge on cytoskeletal and cell adhesion pathway genes, such as *MAPK3*, *MVP* and *KCTD13* (Golzio et al., 2012; Pucilowska et al., 2015; Roth et al., 2020), unleashes the constraints present throughout development, introducing variability and increasing the range of possible outcomes from the developmental course that the cells undertake. In humans, this divergence from neurotypical development should not, in principle, follow predictable routes, but is rather largely dependent on initial starting differences, such as the genetic background of the individual, recessive mutations in the non-deleted homologue or the variability in size of the lost fragment (Pizzo et al., 2019; Coman and Gardner, 2007; Szelest et al., 2021), which translates into the remarkable phenotypic variability observed in individuals with 16p11.2 deletion. Using isogenic cell lines in our ventral organoid model of 16p11.2 deletion eliminates the variability that may arise due to differences in genetic background or due to the size of the deleted fragment. Rather, the deletion of this locus appears to introduce instability into the system, rendering the organoids more sensitive to external culture conditions, as evidenced by how the manifested effects vary between batches but not according to deletion cell lines.

The variability in early growth rates observed in the deletion organoids in this study recapitulates the variable effects of 16p11.2 deletions on sub-cortical MGE and CGE-derived structures reported in the current literature. A recent brain MRI study revealed increased volumes of the basal ganglia structures, putamen and pallidum, in individuals with deletion of the distal 16p11.2 region (Sønderby et al., 2020), whereas another neuroimaging study of the deleted proximal 16p11.2 region reported no alterations in basal ganglia structures (Martin-Brevet et al., 2018). Reports on the incidence of macrocephaly among individuals with the 16p11.2 deletion are equally variable and range from 17% to 69% (Steinman et al., 2016; Qureshi et al., 2014; Zufferey et al., 2012). Therefore, the variability in ventral organoid size reflects the variation in individuals with 16p11.2 deletion and ASD with respect to basal ganglia size and overall brain size. The current reports on the effects of 16p11.2 deletion on cortical NPC proliferation are few and variable, leaving the effects of this CNV on the ventral telencephalon largely unexplored. Enhanced proliferation (Connacher et al., 2022; Pucilowska et al., 2015), no difference in NPC proliferation rates (Deshpande et al., 2017; Roth et al., 2020) or a reduction in NPC proliferation (Urresti et al., 2021), as observed in our ventral progenitors, have been reported, further highlighting the increased variability associated with this deletion.

### Increased rosette organisation suggests premature differentiation in 16p11.2 deletion organoids

Deletion organoids exhibited enhanced rosette formation, which could be a sign of premature differentiation. NPCs progress from unstructured neuroepithelial cells to form rosettes *in vitro* (Ziv et al., 2015) and are capable of responding to different patterning cues to initiate the differentiation into region-specific neuronal fates (Elkabetz et al., 2008). The increased potential of deletion organoids to form rosette might, therefore, render the ventral progenitors more likely to respond better to differentiation signals.

Transcriptomic profiling of cortical neural progenitor cells derived from human iPSCs harbouring the deletion revealed several differentially expressed genes, both within and outside the 16p11.2 locus, involved in cytoskeletal organisation and cell adhesion (Roth et al., 2020). Because rosettes are formed through cytoskeletal events (Harding et al., 2014), we can speculate that the effects of this deletion may converge on cytoskeletal pathways that promote the rearrangement and organisation of ventral progenitors into neural rosettes, without altering the number of neural progenitors within the organoid at earlier stages. Roth et al. (2020) reported no observable differences in the abilities of control and 16p11.2 deletion cortical NPCs to form rosettes, although this finding was not quantified (Roth et al., 2020). In contrast to our findings, the absence of a phenotype in cortical NPCs may suggest that 16p11.2 deletion could affect dorsal and ventral progenitors differently.

COUPTFII is another factor that is an important regulator of differentiation during embryonic development (Polvani et al., 2019) and plays a role in the formation of neural rosettes *in vitro* (Fedorova et al., 2019; Hsu et al., 2017), raising the possibility that increased COUPTFII expression might affect the generation of rosettes in the deletion organoids. Conversely, the higher abundancy of rosettes in the deletion organoids might result in increased COUPTFII expression.

### Prolonged cell cycle length and G1-phase duration drive early differentiation in 16p11.2 deletion organoids

16p11.2 deletion causes ventral progenitors to proliferate more slowly in ventral organoids, which increases their likelihood of undergoing neurogenic cell divisions and differentiating into interneurons. Accelerated ventral telencephalic differentiation might also occur in humans with 16p11.2 deletion, as we observed in ventral organoids. Indeed, many 16p11.2 genes, such as *KIF22*, *ALDOA*, *HIRIP3*, *PAGR1* and *MAZ* were found to be expressed in progenitors and could, therefore, influence neurogenesis (Roth et al., 2020; Morson et al., 2021). To date, only one study used organoids to examine the effect of 16p11.2 deletion on cortical NPC proliferation at 1 month, revealing a decreased proliferation rate and more NEUN^+^ cells (Urresti et al., 2021), which is in line with our findings in ventral organoids.

The increased cell cycle length in the days 33-35 deletion organoids could play a role in the increased NEUN expression observed in day 46 and day 50 deletion organoids. Eventually, the increased withdrawal of cells from the cell cycle and the depletion of the progenitor pool might explain why no differences in NEUN levels were observed between deletion and control organoids at days 70, 90 and 130. During the G1-phase, cells sense different environmental cues to initiate cell-fate decisions (Lanctot et al., 2017; Florio and Huttner, 2014), and its lengthening promotes the transition to a more differentiated progeny (Dalton, 2015; Hwang et al., 2021). The lengthening of G1 phase in cortical progenitors is associated with the fate transition from apical progenitors to basal progenitors, and increases the likelihood that a daughter cell exits the cell cycle, thus promoting neurogenesis (Lim and Kaldis, 2012; Arai et al., 2011; Pilaz et al., 2009; Lange et al., 2009). Labelling experiments in the mouse LGE have demonstrated a progressive lengthening of G1 with embryonic age (Bhide, 1996; Sheth and Bhide, 1997). Thus, the altered cell cycle length and increased NEUN and LHX6 expression in our ventral deletion organoids is compatible with the possibility that this deletion may result in increased interneuron production.

Moreover, recent studies provided evidence for a causal link between the cell cycle length of MGE and LGE progenitors, and the cell fate of their progeny (Zong et al., 2022; Magnani et al., 2010). Our data suggest that this deletion could potentially alter the subtypes of the neuronal progeny, as evidenced by the preferential differentiation towards CLBN1^+^ interneurons over RELN^+^ interneurons. Similar to the findings in our ventral organoids, several post-mortem findings in individuals with ASD reported increased CLBN1 levels and reduced RELN levels in the cerebral and cerebellar cortices (Fetit et al., 2021).

### 16p11.2 deletion accelerates the developmental trajectory of ventral organoids

Collectively, our findings suggest that this deletion results in precocious neurodevelopment, whereby deletion organoids are further along in their development compared with controls. The prolongation of the cell cycle and increased TUJ1 and GAD67 relative mean fluorescence intensity observed in the deletion organoids at days 33-35, followed by increased mean NEUN and LHX6 fluorescence intensity at day 50 suggest earlier birth of interneurons in the ventral organoids carrying the deletion. Unlike LHX6, we observed no reduction in NEUN at 130 days. We speculate that the reduction of NEUN might be observed at even later stages, given that NEUN is expressed in mature neurons, whereas LHX6 is involved in interneuron migration and is expressed in immature neurons. The significant reduction of LHX6 at days 70 and 130 in the deletion organoids further supports the notion that this deletion may cause premature differentiation into interneurons. However, we do not observe a plateau of LHX6 over time, where the control organoids catch up with the accelerated developmental pace of the deletion organoids, suggesting that, in addition to precocious birth of interneurons, other factors might come to play. We further speculate that the significant reductions in NEUN expression between days 90 and 130 in both deletion and control organoids may imply neuronal death over time.

### Conclusions

In conclusion, we propose the following hypothesis based on our findings: at early stages of ventral telencephalic development, the 16p11.2 deletion enhances rosette formation in ventral organoids without altering the proportions of neural progenitors within the organoid. Because neural progenitors organised into rosettes can respond to environmental cues and initiate differentiation into region-specific neuronal fates better than dispersed progenitors, the more-structured NPCs in the deletion organoids are more likely to undergo neurogenic cell divisions. The prolongation of total cell cycle length, as well as the duration of G1 phase also increases the likelihood of ventral progenitors differentiating into interneurons, which eventually becomes evident at later timepoints. Our findings also indicate that the 16p11.2 deletion mechanism introduces more variability into this developing system.

## MATERIALS AND METHODS

### Culturing of iPSC lines

Cells were generated and provided by James Gusella and Derek Tai at the Molecular Neurogenetics Unit, Harvard Medical School (Boston, MA, USA) (Tai et al., 2016). Briefly, isogenic control lines were generated by transfecting the parent iPSC line, GM8, with the Cas9 expression vector lacking any guide RNAs, so as not to induce any genetic modifications. All deletion lines were generated by transfecting the parent iPSC line with the Cas9 expression vector, including the designed guide RNAs to target the homologous sites flanking the 16p11.2 locus. This method generated a 740 kb microdeletion of the 16p11.2 region that mimics the consequences of the *in vivo* non-allelic homologous recombination (NAHR) and mirrors the size of the CNV in humans, as previously described (Tai et al., 2016).

A total of seven iPSC lines were used: the parent line, GM8; three isogenic control lines (FACS51, FACS52 and FACS53); and three deletion lines (DELD5, DELA3 and DELB8). Cells were cultured in feeder-free medium and grown on Matrigel-coated plates in cell medium containing 1:1 mTesR1: Essential 8 (StemCell Technologies, 85850 and Thermofisher A1517001, respectively). The cells were maintained so that once the iPSC colonies were confluent, they could be split and passaged into different wells. The cells were maintained and passaged until stable, growing into healthy colonies and showing very little differentiation before being used to grow organoids.

### Generating ventral organoids

To generate ventral organoids, we adapted the pre-established protocol developed by Sloan et al. (2018). Briefly, once iPSC lines were confluent, differentiated cells were manually removed. Accutase (Stem Cell Technologies, 07920) was added (1 ml/well) and incubated for 5-6 min to detach the cells from the plate. Cells were washed with PBS and resuspended in 1 ml E8:MTESER media with the ROCK inhibitor Y-27632 (10 μM, EMD Chemicals). Nine-thousand cells were seeded per well in a 96-well round-bottomed ultra-Low attachment plate (Corning). The cells remained in E8:MTESER media with ROCK inhibitor for a transition period of 4 days, the medium was replenished once on the second day. This increased the chances of the cells aggregating into organoids. Neuronal induction media (NIM) and neuronal media (NM) were prepared as described previously (Sloan et al., 2018) and summarised in Table S8. After this transition period, individual organoids were transferred from 96-well plates to 24-well plates in NIM supplemented with two SMAD inhibitors; dorsomorphin (5 μM, Sigma-Aldrich) and SB-431542 (10 μM, Tocris), together with the ROCK inhibitor Y-27632 (10 μM, EMD Chemicals). Growing organoids in separate wells, using the same number of cells seeded per well, eliminates the issue of organoid fusion and allows a fair comparison of organoid size. Plates were then incubated at 37° and 5% CO_2_ for 48 h. On the 3rd day, fresh NIM supplemented with dorsomorphin, SB and the Wnt pathway inhibitor IWP-2 (5 μM, Selleckchem) was added and media were changed every day. On day 6, organoids were transferred to NM containing neurobasal A (Life Technologies, 10888), B-27 supplement without vitamin A (Life Technologies, 12587), GlutaMax (1:100, Life Technologies) and penicillin and streptomycin (1:100, Life Technologies), and supplemented with the growth factors EGF (20 ng ml^−1^; R&D Systems) and FGF2 (20 ng ml^−1^; R&D Systems) until day 24. From day 6 onwards, the cells were also incubated on an orbital shaker.

To induce ventral identity, the SHH pathway agonist SAG (smoothened agonist, 100 nM, Selleckchem) was added to NM from day 12 to day 24. IWP-2 (Selleckchem, s7085) and SAG as well as allopregnanolone (100 nM, Cayman Chemicals) were supplemented from day 15 to day 24 with a brief exposure (day 12-15) to retinoic acid (100 nM, Sigma-Aldrich). From day 25 to 42, NM was supplemented with the growth factors BDNF (20 ng ml^−1^, Peprotech) and NT3 (20 ng ml^−1^, Peprotech), and media were changed every other day. From day 43 onwards, organoids were maintained in unsupplemented NM with medium changes every 4-6 days.

### Cryopreservation and immunohistochemistry

Organoids were fixed in 4% paraformaldehyde (PFA) for 30 min to 2 h. They were then washed in PBS three times and transferred to 30% sucrose solution overnight at 4°C. After that, they were transferred into embedding medium containing 1:1 30% sucrose:OCT, snap-frozen on dry ice and stored at −80°C. For immunohistochemistry, 10 μm sections were cut using a cryostat (Leica). Cryosections were warmed at room temperature and washed in running water, followed by washing in 0.1% Triton X-100 diluted in PBS (PBST) to permeabilise the tissue. Sections were then blocked in 10% donkey or goat serum in PBST for 30 min at room temperature. After that, they were incubated overnight at 4°C with primary antibodies diluted in blocking solution, as listed in Table S8. PBST was used to wash off the primary antibodies and the cryosections were incubated with secondary antibodies in blocking solution for 1 h. Finally, nuclei were visualised with DAPI and cryosections were mounted for microscopy on glass slides using Vectashield Hardset (Vector Labs) for imaging on a fluorescent or confocal microscope. Images were processed in ImageJ (Fiji).

### Measuring organoid size

Organoids were imaged in culture using a light microscope at several timepoints across development. Images were then analysed using ImageJ (Fiji). The organoid outline was selected using the polygon selection tool and the area was measured for every organoid image. The individual organoid area was then normalised to the average area of the control organoids in the respective batch. Box plots and line graphs were plotted using R.

### Quantification of neural rosettes

Images were taken at 10× magnification to cover the entire organoid and analysed using ImageJ (Fiji). The organoid outline was selected using the polygon selection tool and the area was measured for every organoid image. Similarly, neural rosettes were outlined, and their total area was measured. The number of neural rosettes per organoid was quantified, together with the total area occupied by the neural rosettes in an organoid. The relative rosette area was also calculated by dividing the total area occupied by neural rosettes in an organoid by the area of the organoid.

### Quantification of COUPTFII expression

Images were taken at 10× to cover the entire organoid and analysed using ImageJ/Fiji. The images were then split into the different channels to quantify the corresponding markers. Cells positive for COUPTFII were manually quantified using the manual cell counter plug-in in ImageJ/Fiji.

### Dissociation of organoids for flow cytometry

Organoids were transferred to six-well plates and washed twice with PBS. Next, the organoids were incubated with 2 ml Accumax (Stem Cell Technologies, #7921) for 10 min at 37°C on an orbital shaker (90 rpm). Using a p1000 tip, organoids were manually dissociated by pipetting up and down then centrifuged at 200 ***g*** for 5 min. The pellet was then resuspended in 1 ml PBS and filtered through a 40 μm filter into 1.5 ml Eppendorf tubes. Cell density and viability were quantified.

### Fixation and permeabilisation of cells

FOXP3 Fix/Perm Buffer set (Biolegend, 421403) was used according to manufacturer's instructions. Briefly, 1 ml of 1× FOXP3 Fix/Perm solution (Biolegend) was added to each tube, vortexed and incubated at room temperature for 20 min. Samples were centrifuged, the supernatant removed then washed once with FACS staining buffer (ThermoFisher, 00-4222-24). Cells were then resuspended in 1 ml 1× Biolegend's FOXP3 perm buffer and incubated in the dark at room temperature for 15 min. After that, the samples were centrifuged and resuspended in 100 μl of 1× FOXP3 Perm buffer. A master mix of the primary conjugated antibodies for SOX2, Ki67 and TUJ1, together with Hoechst 33342 was prepared in FACS staining buffer (Table S8) and the cells were incubated with the master mix for 1 h in darkness at room temperature. Single staining controls were prepared for every antibody used as well as an unstained control. Cells were washed once with FACS staining buffer and resuspended in 300-500 μl FACS staining buffer for analysis with the flow cytometer. Samples were analysed using the BD LSRFortessa cell analyser at the QMRI flow cytometry and cell-sorting facility (University of Edinburgh).

### Flow cytometric analysis

Data were processed using FlowJo v10.7.2. Briefly, single-stained and unstained controls were used to identify and set thresholds for our gates. A combination of density plots and contour maps for forward scatter (FSC) was used to clearly outline the cell populations positive for the individual markers, particularly where no clear single histogram peaks could be identified. Doublets were excluded: first, by plotting Hoechst-Area versus Hoechst-Height to eliminate G0 and G1 doublets from the rest of the cells in the cell cycle, and then using the FSC-Area versus FSC-Height to further confirm that only single cells were included. From the parent single cell population, TUJ1^+^ neuron and SOX2^+^ progenitor populations were then identified and quantified. The proportion of SOX2+Ki67+ cells (proliferative pool) were also quantified from the population of SOX2^+^ cells. From the SOX2^+^ progenitors, cells positive for Ki67 were plotted against Hoechst 33342 stained cells to differentiate the cells based on their DNA content and quantify the number of cells in the different cell cycle phases (Fig. S6). Finally, results were exported to be processed in R.

### IdU/BrdU double labelling

IdU/BrdU double labelling was carried out as described previously (Martynoga et al., 2005) by sequentially exposing the organoids to the halogenated thymidine analogues iododeoxyuridine (IdU) and bromodeoxyuridine (BrdU), which are incorporated into the synthesised DNA during S phase. Briefly, IdU and BrdU labelling solutions were freshly prepared in IdU/BrdU solvent (0.9% NaCl). Organoids were transferred to a 5 cm dish. A first pulse of 100 μM IdU labelling solution was added to 5 ml of the media and incubated for 1.5 h at 37°C and 5% CO_2_ on an orbital shaker (45 rpm). This labels all the progenitors in S phase (S_cells_) at the beginning of our experiment. After 1.5 h, the organoids are then exposed to a pulse of 100 μM of the BrdU labelling solution and incubated on an orbital shaker for 30 min to make sure that all BrdU was well incorporated into the progenitors then fixed for analysis. This labels S_cells_ at the end of the experiment. Because neural progenitors are not synchronised as they go through the cell cycle (Takahashi et al., 1993), the initial cohort of IdU-labelled cells will exit S phase at a constant rate and will constitute the fraction of leaving cells (L_cells_) that are positive for only IdU and not BrdU. In this experimental set-up, the time interval during which the cycling progenitors can incorporate only IdU (Ti) is 1.5 h. Finally, organoids were fixed for staining and processed for immunohistochemistry analysis as described above. Using antibodies that allow for the differentiation between cells labelled with IdU only and those labelled with both IdU and BrdU (Table S8), we quantified the L_cells_ and S_cells_.

### Quantification of cell cycle kinetics

Calculation of the total cell cycle length (TC) and the duration of G1 (T_G1_), S (Ts) and G2M (T_G2M_) phases was carried out as described previously (Martynoga et al., 2005; Mi et al., 2013). Briefly, because it has been shown that the ratio of the length of an individual phase of the cell cycle to that of another phase equals the ratio of the cell numbers in the two phases (Nowakowski et al., 1989), the ratio between T_i_ (1.5 h) and T_s_ (duration of S phase) equals the ratio between L_cells_ (IdU^+^BrdU^−^) and S_cells_ (IdU^+^BrdU^+^) as shown below:
(1)

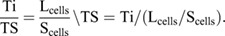
The fractions of L_cells_ (IdU^+^BrdU^−^) and S_cells_ (IdU^+^BrdU^+^) were first quantified and T_s_ was calculated as per Eqn 1. Table S5 lists the numbers of L_cells_ and S_cells_ calculated from the double IdU/BrdU labelling experiment for the organoids analysed in the different cell lines across the four batches. The number of proliferating progenitor cells, which we refer to as the proliferative pool (SOX2+Ki67+) and calculated from flow cytometry, was denoted as P_cells_. Table S6 summarises the average numbers of P_cells_, together with the average number of cells in G1, S and G2M phases calculated from all organoids analysed using flow cytometry.

The total cell cycle length (T_c_) can then be calculated using the ratio between T_s_ and T_c_, which equals the ratio between S_cells_ and P_cells_, as shown below:
(2)

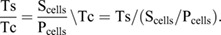
Having already calculated T_s_ from the double-labelling experiment using Eqn 1, we used the values of S_cells_ and P_cells_ calculated from the flow cytometry (Table S6) to calculate TC using Eqn 2. Once T_c_ was calculated, the duration of cells in G1 (T_G1_) was calculated as per Eqn 3:
(3)

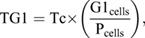
where G1_cells_ is the number of cells in G1 phase and P_cells_ is the number of cycling cells in our progenitor pool. Dividing G1_cells_ by P_cells_ gives us the fraction of cells in G1 phase. Multiplying this value by total cell cycle length provides us with the duration of G1 (T_G1_). Finally, having calculated Tc, Ts and T_G1_, we were able to calculate T_G2M_ by simple subtraction, as shown in Eqn 4:
(4)

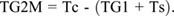


### RNA extraction

Organoids were loaded onto the QIAshredder (Qiagen, 79654) homogeniser to homogenise the tissue and the lysate is then collected. RNA was then extracted using RNeasy Mini Kit (Qiagen, 74104) according to manufacturer's instructions. RNA was eluted in a total of 20 μl RNase-free water. The extracted RNA was quantified using Nanodrop and prepared for real-time quantitative polymerase chain reaction (RT-qPCR).

### Real-time quantitative PCR (RT-qPCR)

cDNA was prepared using LunaScript RT SuperMix Kit (New England Biolabs, E3010L), together with no-RT control reaction and no-template controls, according to manufacturer's instructions. Reactions were then incubated in the thermocycler at 25° for 2 min for primer annealing, at 55° for 10 min for cDNA synthesis and finally at 95° for 1 min for heat inactivation. cDNA was then diluted to a final concentration of 1 ng/µl or 1 ng/10 µl reaction for samples with lower concentrations. RT-qPCR was performed using the Luna Universal qPCR Master Mix. Primers sequences are listed in Table S8 and are adapted from Birey et al. (2017). The ΔΔCt method was used to normalise and quantify relative fold changes in gene expression to two housekeeping genes.

### Quantification of mean fluorescence intensity

Images were taken at 5× magnification to cover the entire organoid and analysed using ImageJ/Fiji. Sections were selected to represent the middle of the organoid with consistent cell densities. The images were then split into the different channels to quantify the corresponding markers. The polygon tool was used to delineate the organoid and measure its area. The mean grey value was measured to represent the mean intensity of the cells expressing the marker of interest within the organoid. The relative intensity was calculated by dividing the mean fluorescence intensity by the organoid area.

### Data analysis and statistics

Analysis was performed using R studio (versions 3.3.2 and 4.0.4). Graphs were plotted using the R package (ggplot2). Levene's Test in the R package (car) was used to assess the homogeneity of variance. Because our data did not fulfil the homoscedasticity assumption, non-parametric Welch's ANOVA was used to assess statistical significance. Post-hoc comparisons were performed using Games Howell test, an alternative to Tukey's comparisons, in the R package (rstatix). Finally, the effect size was calculated using the non-parametric Cohen's d function in the R package (effsize).

Statistical analysis was performed on the flow cytometric dataset using linear mixed effects (LME) modelling to account for the variation introduced into our findings due to batch effects. This was followed by type III ANOVAs to attain *P*-values and assess the statistical significance of LME models. The models generated were both random intercept models, to account for baseline-differences in batches, and random slope models, to accommodate the effect of different cell lines potentially being different for different batches. The R package (lmerTest) was used to design and generate LME models. The R package (car) was then used to run ANOVA tests on our models to assess the statistical significance of our findings. Finally, the R package (sjstats) was used to calculate the effect sizes using omega_squared function.

## Supplementary Material

10.1242/develop.201227_sup1 Supplementary information

## People behind the papers

This article has an associated People behind the papers interview with some of the authors.
