# Supplementary material for: 16p11.2 deletion accelerates subpallial maturation and increases variability in human iPSC-derived ventral telencephalic organoids
Source: Development. 2023 Feb 24;150(4):dev201227. doi: 10.1242/dev.201227 (PMC10110424; doi:10.1242/dev.201227)
Supplement: Supplementary information [file develop-150-201227-s1.pdf]

# Fig. S1

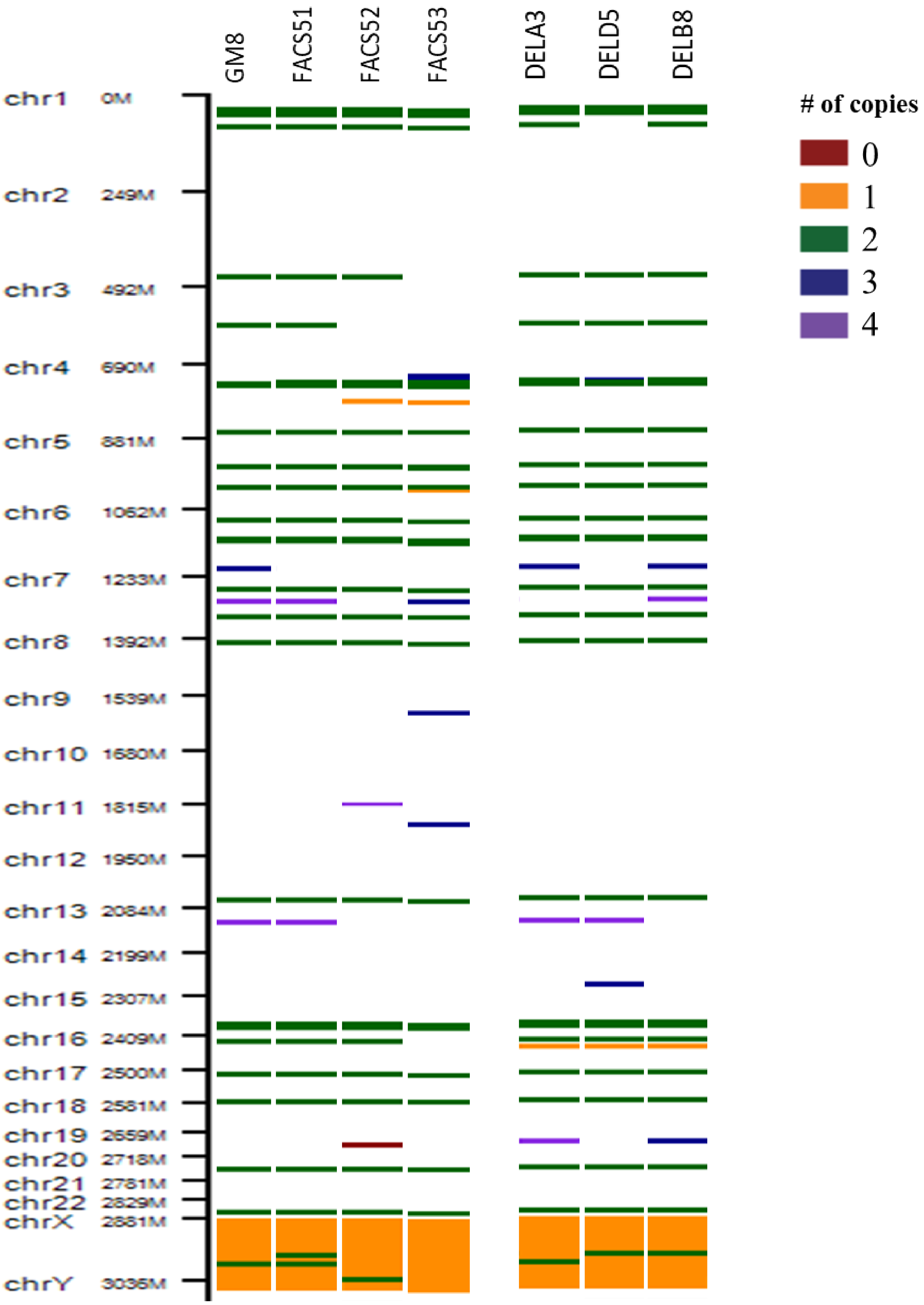


Development • Supplementary information

**Fig. S1. CytoSNP array analysis of 16p11.2 deletion and control lines.** Figure shows the CNV region display generated by Genomestudio outlining the different CNVs present in our iPSC lines across the different chromosomes. The banding patterns are shown for the four control lines: GM8, FACS51, FACS52 and FACS53. GM8 is the parent line from which all other lines were derived. FACS51, FACS52 and FACS53 are the isogenic control lines. The deletion lines are shown to the right. DELD5 and DELA3 were used in the first and second parts of the study, whereas DELB8 was used in the second part of this study. The legend shows how the colour of the bands corresponds to the number of copies present in the CNV region: 0 copies (red) represent a homozygous deletion, 1 copy (orange) represents a heterozygous deletion, 2 copies (green) represent a copy neutral loss of heterozygosity (LOH) mutation, 3 copies (blue) represent a heterozygous duplication and 4 copies (purple) represent a homozygous duplication. The parent line, GM8, was obtained from a male subject, hence the orange band at the Y chromosome. All deletion lines contained the 16p11.2 deletion locus (yellow arrow).

# Fig. S2

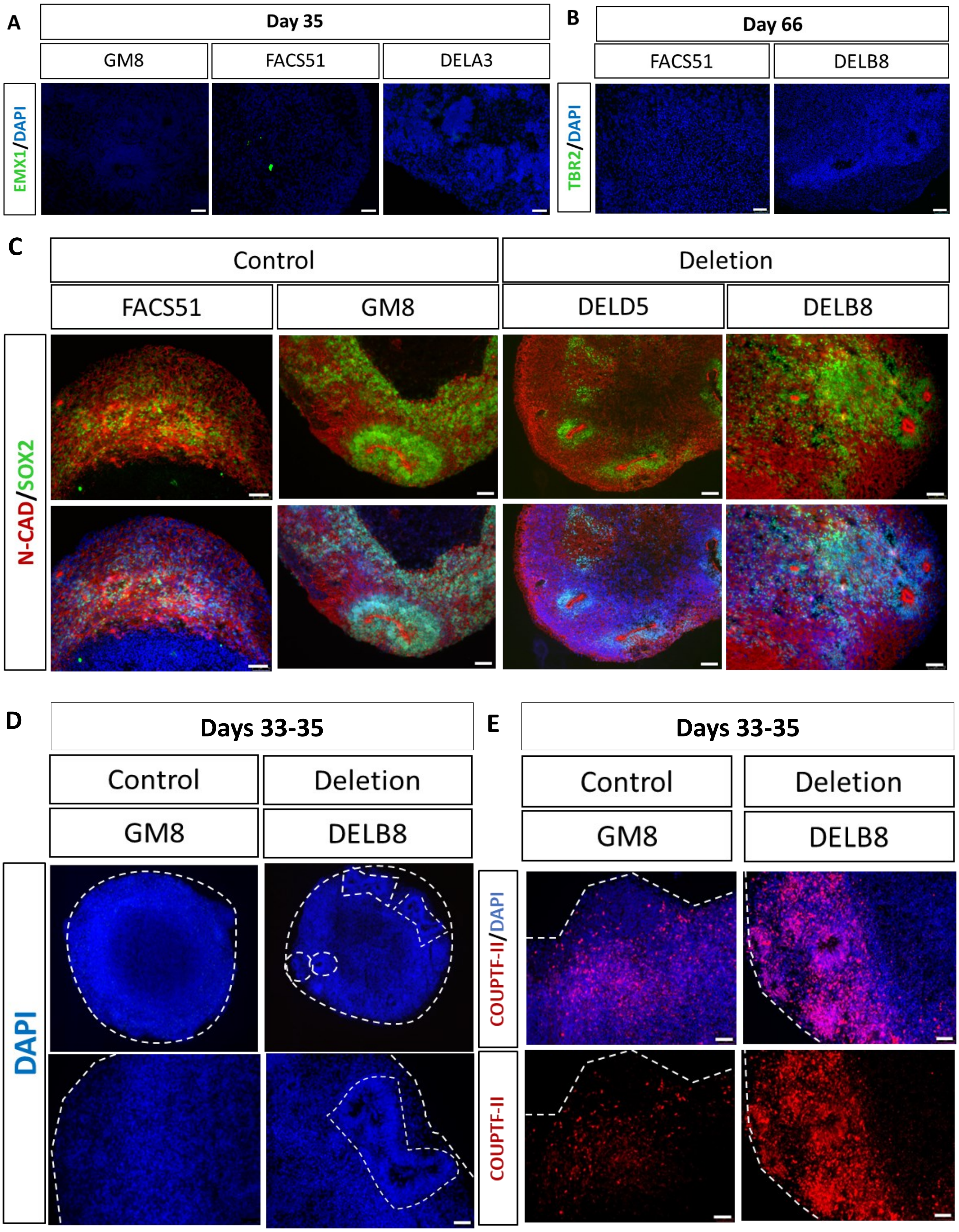


Development • Supplementary information

**Fig. S2. Assessment of dorsal identity in ventral organoids.** (A,B) Representative images of ventral organoids from several control and deletion lines showing no expressing of the dorsal markers EMX1 and TBR2 at days 35 and 66, respectively. Scale bar = 50µm. (C) N-CAD and SOX2 expression in organoids from representative control and deletion lines at day 35. No differences in rosette morphology and arrangement of NPCs around the inner lumen were observed between deletion and control organoids. Organoids from control lines did not always form rosettes, as shown in FACS51. Scale bar = 50 µm. (D) Rosettes in representative ventral organoids at days 33-35 from control and deletion lines used in part 3 to assess differentiation. Scale bars = 25 and 50µm. Organoid perimeter and rosettes are outlined in dashed lines. (E) COUPTFII expression in control and deletion lines used in part 3. Top panels show COUPTFII and DAPI, bottom panels show the red channel for COUPTFII only. Scale bar = 50 µm.

Development • Supplementary information

# Fig. S3

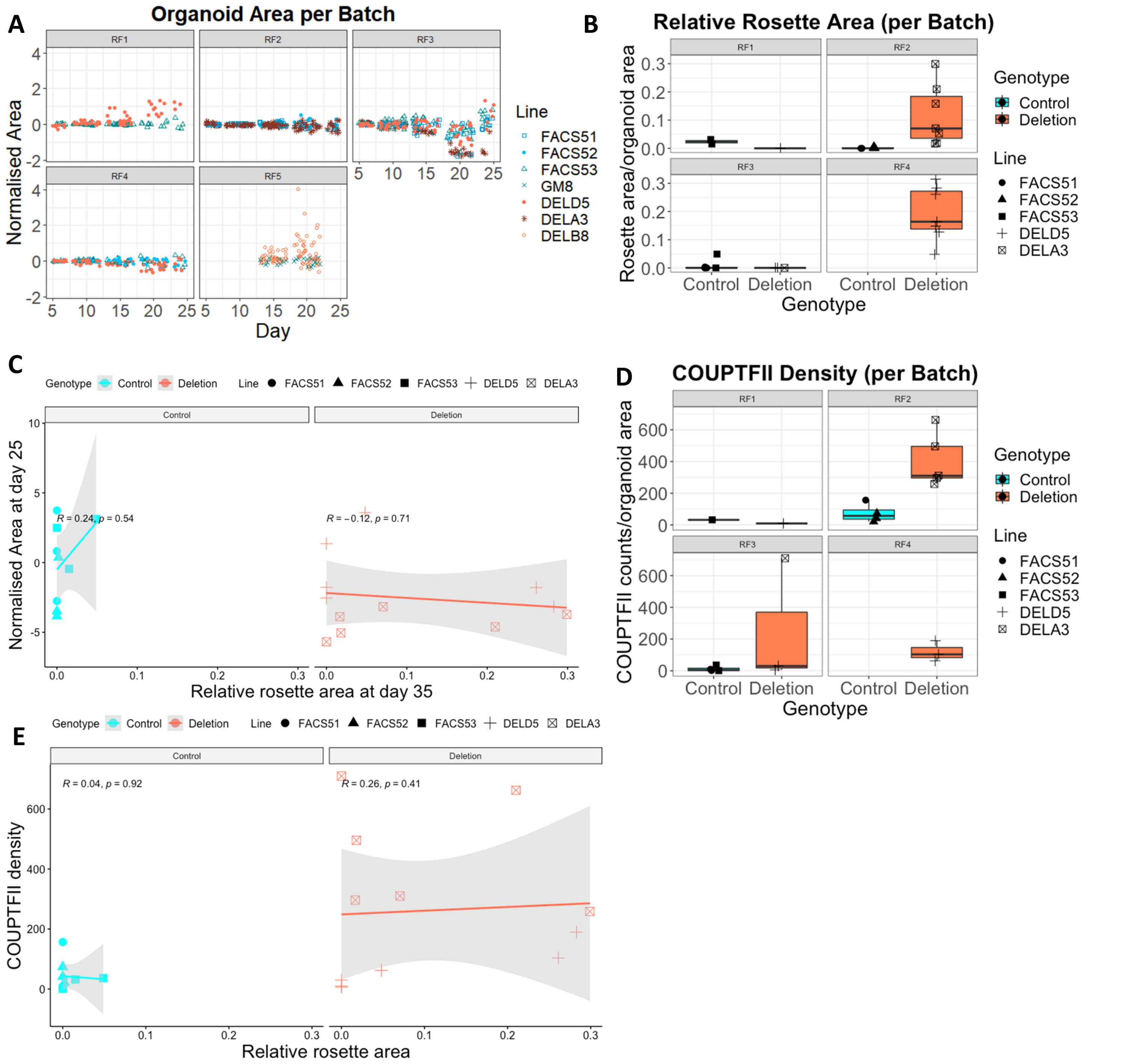


**Fig. S3. Findings grouped by batches and correlation analysis.** (A) Organoid area is normalised for the average control of the corresponding batch (Normalised Area). The data for the different batches (RF1-5) used across the three parts of this study are shown separately. Control organoids (blue) in each batch grew at relatively similar rates, whereas deletion organoids (red) grew at more variable rates. DELD5 organoids grew larger than control organoids in Batch RF1, whereas they grew at a similar rate to organoids of both control lines in Batch RF4. DELA3 organoids grew at a similar rate to control organoids in Batch RF2 but grew slower in Batch RF3. LME analysis considers such batch-batch variability when assessing statistical significance. (B) Boxplots showing the relative rosette area for the organoids in the different batches. Deletion organoids from both lines have the potential to form rosettes that occupy larger organoid area as seen in DELA3 and DELD5 organoids from batches RF2 and RF4 respectively. Compared to controls, the relative rosette area in deletion organoids is more variable. Control samples in the batch RF4 were lost during cryosectioning. (C) Correlation analysis between relative rosette area at day 35 and normalised organoid size at day 25. No significant correlation observed. (D) COUPTFII density (COUPTFII counts normalised to the organoid area) shown per batch. (E) Spearman correlation analysis between relative rosette area and COUPTFII density grouped by genotype at days 33-35. Every point is an individual organoid and different shapes represent the different cell lines used. A moderate correlation is observed which did not reach statistical significance in deletion organoids (Spearman correlation, R=0.26, p=0.41).

Development • Supplementary information

# Fig. S4

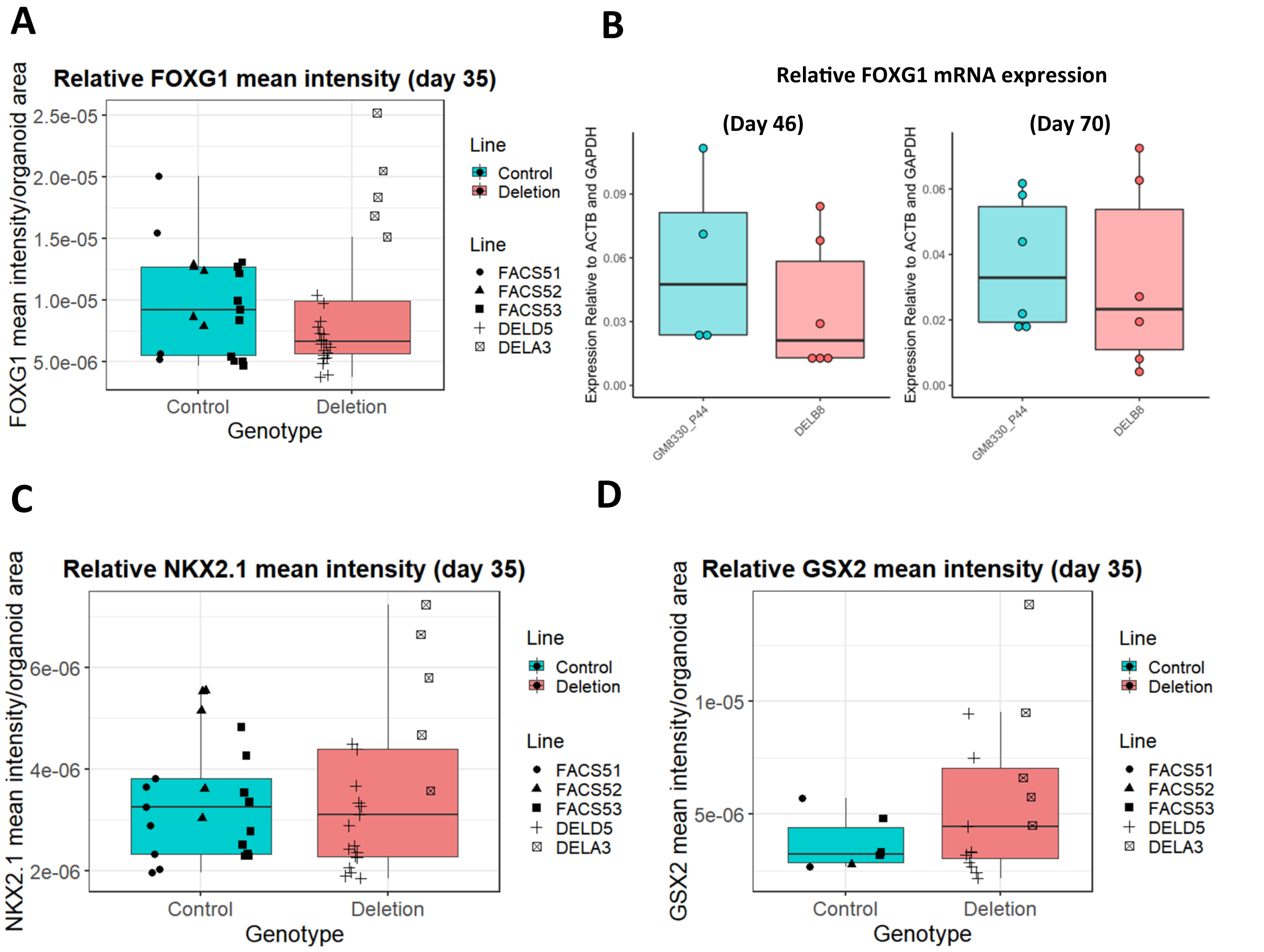


**Fig. S4. Quantification of forebrain and ventral telencephalic marker expression using IHC and RT-qPCR.** (A) Mean FOXG1 fluorescent intensity relative to organoid size quantified from IHC sections at day 35. No significant differences observed between deletion and control organoids (Welch Two Sample t-test, p = 0.6645). (B) Quantification of the relative FOXG1 mRNA expression at days 46 and 70 by RT-qPCR. No significant differences found between deletion and control organoids, suggesting no differences in the ability to acquire forebrain identity between the two genotypes. (C,D) Mean NKX2.1 and GSX2 fluorescent intensity relative to organoid size quantified from whole-organoid IHC sections at day 35. No significant differences observed between deletion and control organoids (Welch Two Sample t-test, p = 0.8427 and 0.1059 respectively).

Development • Supplementary information

# Fig. S5

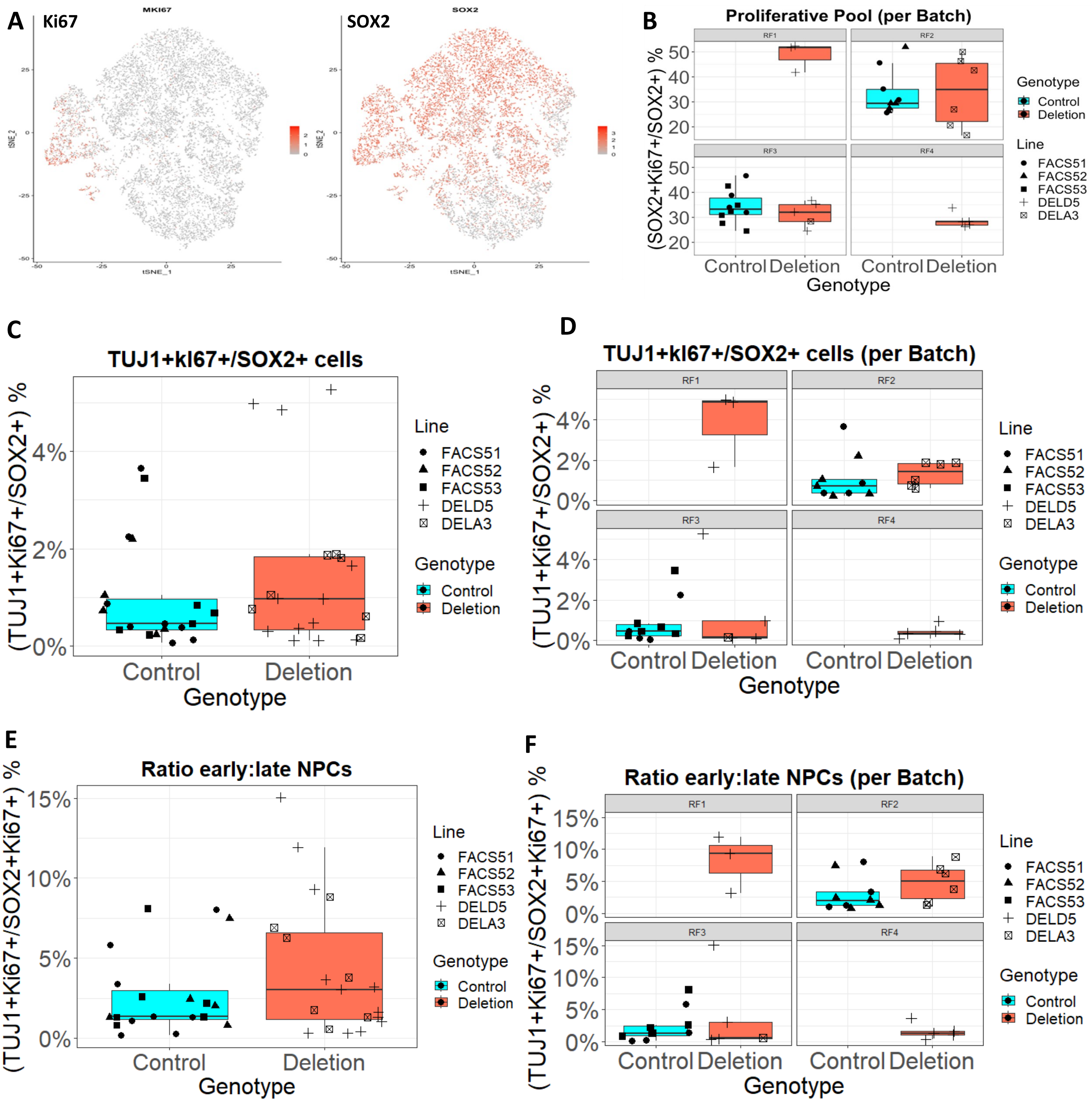


Development • Supplementary information

**Fig. S5. Analysis of proliferation and differentiation in ventral organoids.** (A) Re-analysis of Ki67 (MKI67, left) and SOX2 (right) expression in published single cell RNA-seq dataset of ventral organoids at day 30 (GEO accession number: GSE97882; Xiang et al., 2017). Normalisation, dimensionality reduction and clustering of single cells were performed in R using the computational software package Seurat (v3.0) as described by Xiang et al., 2017. Briefly, to improve downstream dimensionality reduction and clustering, linear transformation was applied to scale the data and principal component analysis (PCA) was then conducted using the ScaleData and RunPCA functions respectively. The functions JackStrawPlot and ElbowPlot were used to determine statistically significant principal components (PCs). The first 20 PCs were included in this analysis. Cells were clustered using the FindClusters function with a resolution of 0.5. We then performed dimensionality reduction by t-Distributed Stochastic Neighbour Embedding (t-SNE). Cells expressing Ki67 or SOX2 are shown in red. Not all SOX2 cells are cycling, only a small proportion of cells express the cell cycle marker Ki67, similar to our findings from flow cytometry. (B) Percentage of the proliferative pool (SOX2 +Ki67+/SOX2+) grouped by batch. 17/57 organoids were excluded on the basis that very few SOX2 +Ki67+ cells remained after doublet exclusion to perform rigorous cell cycle analysis. Excluded sample size by genotype: Control n=11, Deletion n=7. Excluded sample size by cell line and batch: FACS51 n=1 in batch RF3. FACS52 n= 3 in batch RF4. FACS53 n=7 (2 in batch RF1, 1 in batch RF3 and 4 in batch RF4). DELA3 n=3 (1 in Batch RF2, 2 in batch RF3). DELD5 n=4 (1 in batch RF3, 3in batch RF4). (C,D) Proportion of TUJ1+Ki67+ late NPCs in ventral organoids expressed as percentage of SOX2+ cells in the organoid grouped by genotype and by batch respectively. (E,F) Ratio of late proliferating NPCs (TUJ1+Ki67+) to early proliferating NPCs (SOX2+Ki67+) in ventral organoids grouped by genotype and by batch respectively. Sample size by genotype: n= 21 organoids for control and deletion. Sample size by cell line: FACS51 n=9, FACS52 n=5, FACS53 n=7, DELD5 n=14, DELA3 n=7.

# Fig. S6

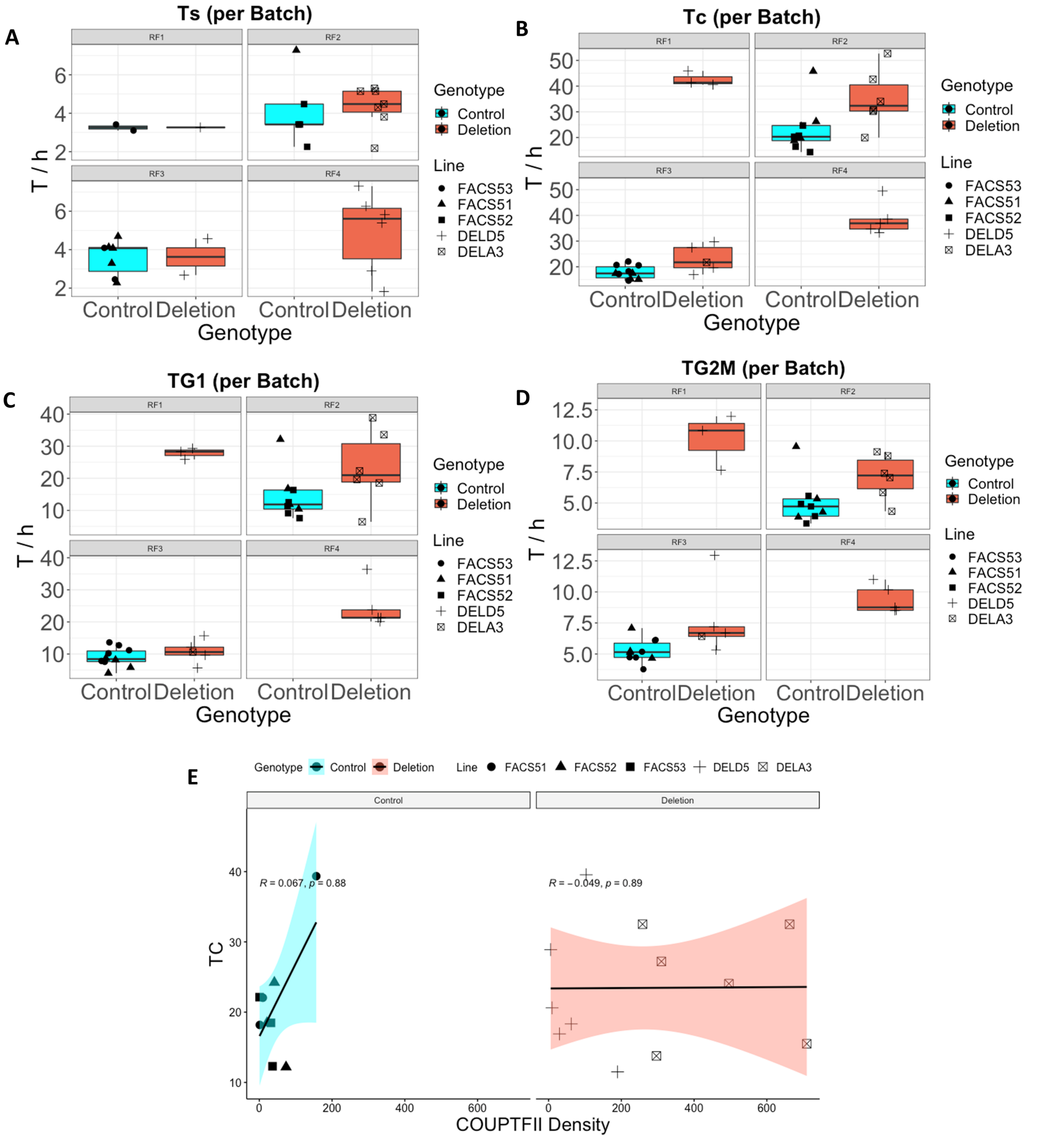


**Fig.S6. Cell cycle kinetics grouped by batch and correlation Analysis.** (A) Duration of S-phase calculated from double IdU/BrdU labelling experiment grouped by batch. (B-D) TC, TG1 and TG2M calculated using the number of cells in the individual phases from flow cytometric analysis grouped by batch. (E) Correlation analysis between TC and COUPTFII density in the imaging dataset (Spearman correlation).

# Fig. S7

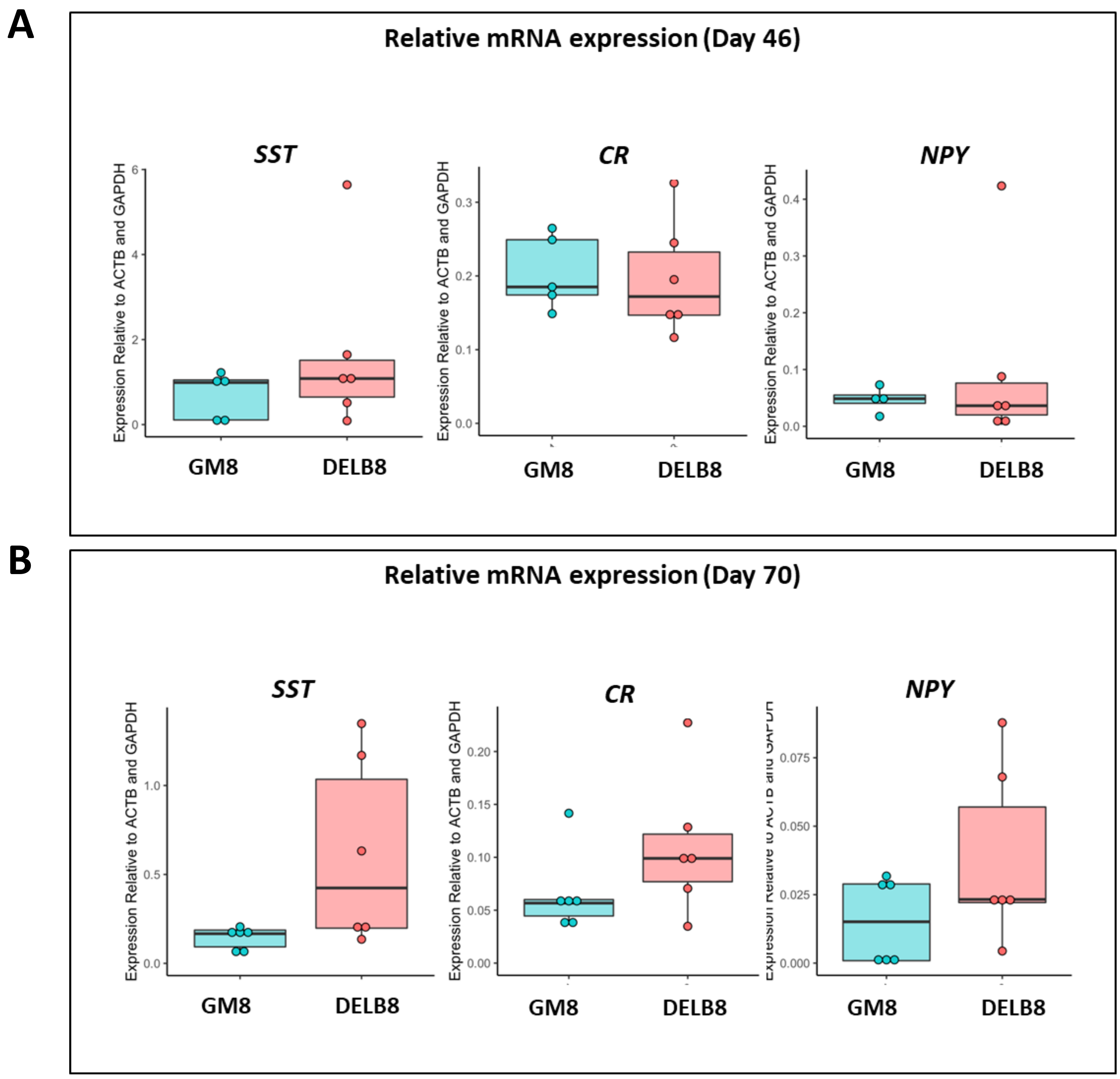


**Fig. S7. RT-qPCR analysis of additional interneuron markers at days 46 and 70 in the lines GM8 and DELB8.** (A,B) Relative mRNA expression the interneuron markers Somatostatin (*SST*), Calretinin (*CR*) and Neuropeptide-Y (*NPY*) at days 46 and 70 respectively by RT-qPCR.

Development • Supplementary information

# Fig. S8

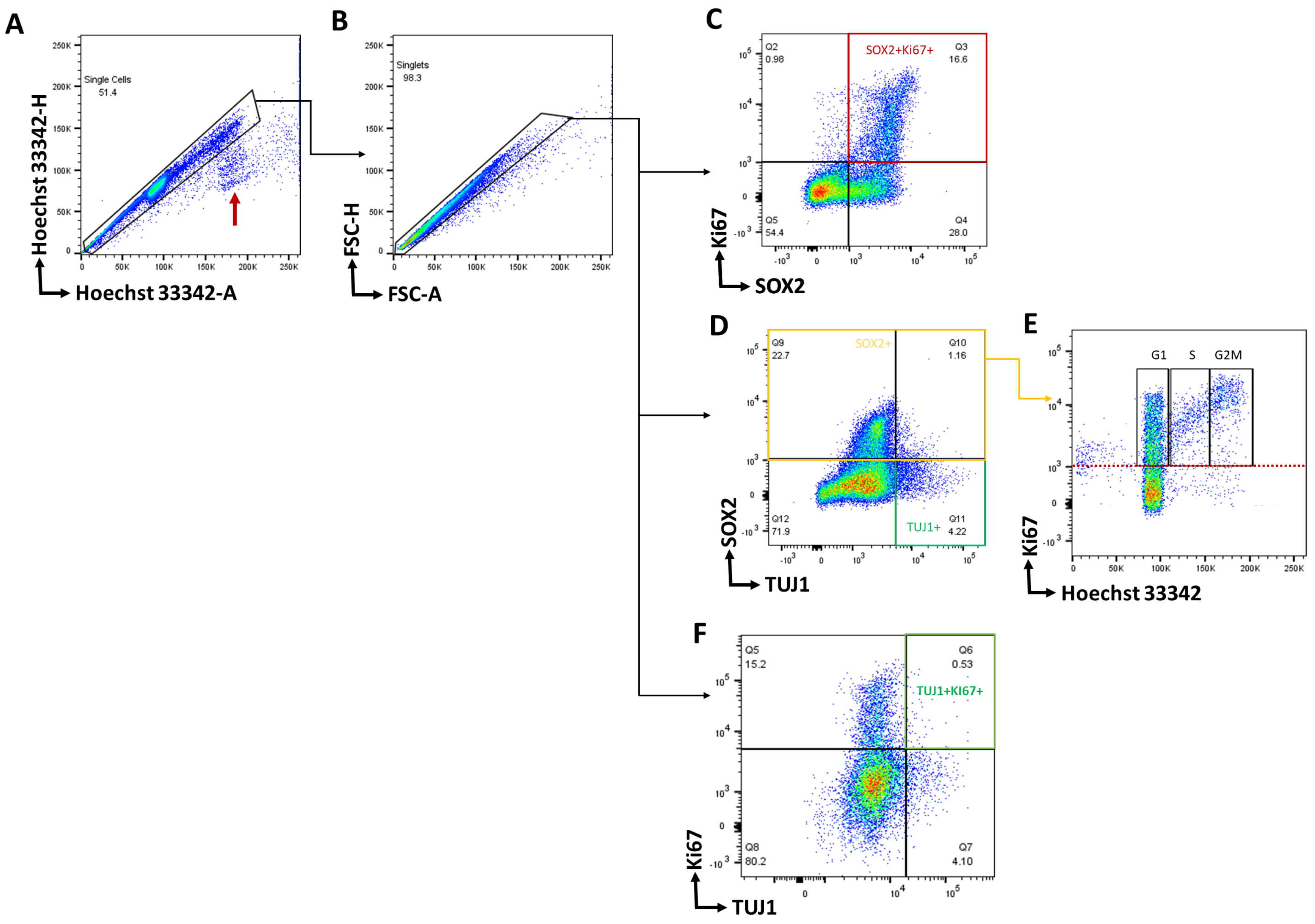


**Fig.S8. Gating strategy to isolate different cell populations in ventral organoids using flow cytometry.** Cells were labelled with Hoechst 33342 and antibodies for Ki67, SOX2 and TUJ1. (A) Doublet exclusion using Hoechst-A (Area) against Hoechst-H (Height). Red arrow shows G0/G1 doublets excluded from cell population. G0/G1 doublets have the same height as G0/G1 single cells but double the area. (B) FSC-A against FSC-H to further exclude any remaining doublets from the parent population in A. This is our final single-cell population that is used in the downstream analysis. (C) Density plot showing SOX2-A on the x-axis against Ki67-A on the y-axis to isolate all the SOX2+ki67+ cycling progenitors which we refer to as the proliferative pool (red box). In this representative sample, 16.6% of the single cells (singlets in B) were double positive for SOX2 and Ki67 (Top right quadrant). (D) Density plot showing TUJ1-A on the x-axis against SOX2-A on the y-axis. In this representative sample, 4.22% of the single cells (singlets in B) were TUJ1+ (bottom right quadrant). (E) Density plot showing Ki67 against Hoechst. All SOX2+ cells (Yellow box in D) were used to isolate SOX2+ cells in the cell cycle that are also positive for Ki67 (above the dotted red line). (F) Density plot showing TUJ1-A on the x-axis against Ki67-A on the y-axis to isolate all the TUJ1+Ki67+ cycling late NPCs (green box).

Development • Supplementary information

**Table S1. Study Design**

Click here to download Table S1

**Table S2. CNV Analysis**

Click here to download Table S2

**Table S3. Organoid Area Analysis**

Click here to download Table S3

**Table S4. Post-hoc Comparisons**

Click here to download Table S4

**Table S5. All findings for organoids in IHC dataset**

Click here to download Table S5

**Table S6. All findings for organoids in flow cytometry dataset**

Click here to download Table S6

**Table S7. NEUN and LHX6 measurements at days 50, 90 and 130**

Click here to download Table S7

**Table S8. List of Reagents, antibodies and primers used in this study**

Click here to download Table S8

**Table S9. Quantification of Late NPCs (TUJ1+Ki67+) in Flow cytometry**

Click here to download Table S9

**Table S10. Quantification of forebrain and ventral telencephalic markers at day 33-35**

Click here to download Table S10

**Table S11. Quantification of relative mean fluorescent TUJ1 and GAD67 intensity at day 33-35**

Click here to download Table S11
