# Supplementary material for: 16p11.2 deletion accelerates subpallial maturation and increases variability in human iPSC-derived ventral telencephalic organoids
Source: Development. 2023 Feb 24;150(4):dev201227. doi: 10.1242/dev.201227 (PMC10110424; doi:10.1242/dev.201227)
Supplement: Supplementary file 1 [file develop-150-201227_review_history.pdf]

16p11.2 deletion accelerates subpallial maturation and increases variability in human iPSC-derived ventral telencephalic organoids

Rana Fetit, Michela Ilaria Barbato, Thomas Theil, Thomas Pratt and David J. Price
DOI: 10.1242/dev.201227

Editor: Francois Guillemot

Review timeline

Original submission:	23 August 2022
Editorial decision:	3 October 2022
First revision received:	13 December 2022
Editorial decision:	9 January 2023
Second revision received:	16 January 2023
Accepted:	19 January 2023

Original submission

First decision letter

MS ID#: DEVELOP/2022/201227

MS TITLE: 16p11.2 locus decelerates subpallial maturation and limits variability in human iPSC-derived ventral telencephalic organoids

AUTHORS: Rana Fetit, Thomas Theil, Thomas Pratt, and David Price

I have now received the reports of three referees on your manuscript and I have reached a decision. The referees' comments are appended below, or you can access them online: please go to BenchPress and click on the 'Manuscripts with Decisions' queue in the Author Area.

As you will see, all the referees are enthusiastic about your work, but they also have significant criticisms and recommend a substantial revision of your manuscript before we can consider publication. In particular, they criticise the use of different mutant/control cell lines for the early stages and late stages analyses; they recommend that you include markers of dorsal telencephalic identity, as well as non-neural markers, in your analysis; and they also suggest that you investigate the timing of neurogenesis in the mutant organoids. If you are able to revise the manuscript along the lines suggested, which may involve further experiments, I will be happy to receive a revised version of the manuscript. Your revised paper will be re-reviewed by the original referees, and its acceptance will depend on your addressing satisfactorily all their major concerns. Please also note that Development will normally permit only one round of major revision.

If it would be helpful, you are welcome to contact us to discuss your revision in greater detail. Please send us a point-by-point response indicating your plans for addressing the referee’s comments, and we will look over this and provide further guidance.

Please attend to all of the reviewers' comments and ensure that you clearly highlight all changes made in the revised manuscript. Please avoid using 'Tracked changes' in Word files as these are lost in PDF conversion. I should be grateful if you would also provide a point-by-point response detailing how you have dealt with the points raised by the reviewers in the 'Response to Reviewers' box. If you do not agree with any of their criticisms or suggestions, please explain clearly why this is so.

© 2023. Published by The Company of Biologists under the terms of the Creative Commons Attribution License (https://creativecommons.org/licenses/by/4.0/).

Reviewer 1

Advance summary and potential significance to field

Autism Spectrum Disorder (ASD) can arise due to microdeletions in 16p11.2 but it is not yet clear what effect this has on the developing brain and thus the pathogenic mechanisms are still unclear. Previous work with cortical organoids harboring 16p11.2 CNVs has shown abnormal progenitor behavior with excess neurogenesis. However, these previous models only looked at excitatory neurogenesis and did not examine production of inhibitory neurons. Fetit et al. have now examined exactly this by using directed differentiation of organoids towards ventral telencephalon, and hence toward the inhibitory lineage. The authors find that, like in 16p11.2 associated autism, there is more variability in overall size, and that progenitors exhibit abnormal cell cycle and neurogenesis. This is an interesting finding and has important implications for the field of ASD.

Comments for the author

I do have several concerns regarding the interpretation, and the still rather unclear conclusions which would benefit from a few specific experiments. These comments are detailed below and I hope they will help the authors improve the manuscript.

Major comments:

1. The fact that multiple isogenic cell lines were used is elegant and powerful, and the careful genetic analysis of the lines to confirm genetic status is important. In addition, the use of multiple batches with different cell lines represented across at least two batches, particularly for the early stage analysis, is an excellent experimental design. I do however have a concern regarding the latest stage analysis (Day 50-70), and the fact that a completely different set of control and mutant cell lines were used, and it was only done in one batch. Given line-to-line variability, and batch-to-batch variability, in order to draw conclusions across the stages, it would be good to include a pair of control and mutant cell lines from the earlier stages (ideally the pair with the least off-targets) also in the latest stage analysis in order to connect the findings.

2. Regarding identity of the generated organoids, it would be good to also check for dorsal cortical identity, given that the “default” is dorsal identity and thus it would be helpful to explain some of the variability. In addition how effective was neural identity acquisition? The somewhat disorganized tissues suggest nonneural tissue. Could there be issues with differentiation towards neuroepithelium? Markers of mesoderm or neural crest identities would be good to see here. The issue of identity would also be an important one given the effects later described on COUPTFII levels.

3. Regarding overall size, it is interesting that early on there is a lot of variability in size of the mutant organoids. Could this reflect different rates of the progress of the effect on neurogenesis? In other words, what happens to overall organoid size at much later stages? Do the mutants actually end up being larger or smaller than control when analyzed at even later stages? It would be interesting to see what size the organoids are at very late time points beyond day 70 ideally.

4. Regarding cell cycle analysis, the fact that lengthened cell cycle is correlated with larger rosette size is somewhat counterintuitive to what might be expected. Longer cell cycle should result in fewer cells over the same given amount of time, and thus smaller tissues. In addition, cell cycle lengthening of progenitors is known to increase as neurodevelopment proceeds, so you might expect that progenitors with longer cell cycles would be less neuroepithelial and have less rosette potential. Could this actually reflect a difference in stage? Could it be that mutants are further along in their development relative to absolute time? It would be helpful to look at other readouts of neurogenic stage. For example, when does neurogenesis begin?

5. Regarding the overall interpretation of the findings, and the model, the data seem to point to precocious neurodevelopment: longer cell cycle is associated with later stage neural progenitors, presence of increased NeuN which marks mature neurons and could actually reflect earlier birth of neurons, thus giving them more time to mature and express NeuN. Similar to the point above, it would be helpful to know when neurons begin to be produced and whether that is earlier in mutant organoids. Precocious development would also be expected to eventually lead to fewer

© 2023. Published by The Company of Biologists under the terms of the Creative Commons Attribution License (https://creativecommons.org/licenses/by/4.0/).

interneurons at the end. This might be what is beginning to happen at day 70, and even later the mutants may even have fewer NeuN+ neurons. Thus a very late stage examination would be helpful and this could be combined with a size measurement as raised above.

Minor comments:
1. The title is a bit confusing, and actually I found the running title more straightforward. I would prefer a title that directly states what was done (deletion of 16p11.2) and the result.
2. What is meant by “replicates” within batches?

Reviewer 2

Advance summary and potential significance to field

The manuscript by Fetit et al., uses ventral telencephalic organoids to study the effect of the 16p11.2 microdeletion on interneuron development. They found that deletion organoids were more variable in size, had increased and more variable rosette area and a lengthening of G1 phase with premature differentiation into neurons. Overall, the manuscript addresses an important and under-explored aspect of the 11p11.2 microdeletion.

Comments for the author

The manuscript is a pleasure to read. It is well written, it provides sufficient background with references, and it states the question in a clear manner. Experiments are well designed, and figures are generally well presented. The use and characterization of hiPSCs is also quite good. Authors performed a thorough characterization of the lines used, clearly explained how many lines/batches were used per experiment, and transparently showed data in each experiment separated by batch.

With this in mind, there are a few additional experiments that I believe would strengthen the manuscript with specific emphasis on ventral cells and interneurons and the hiPSC lines used:

1. Some additional data on the fate and specification of the ventral organoids would be helpful to interpret the results. Specifically, although the authors show immunohistochemistry for some ventral markers in Figure 1, it is important to show that the fate of the deletion organoids is not changed compared to controls. If immunohistochemistry quantification is not a possibility, qPCRs for ventral forebrain markers at the early time point, as well as markers for other CNS regions should be performed.

2. The authors conclude in the Abstract: “We propose that 16p11.2 microdeletions increase developmental variability and may contribute to ASD aetiology by lengthening the cell cycle of ventral progenitors, promoting premature differentiation into interneurons”. Quantification in Figure 5 showing interneuron markers in addition to NeuN would be important to support this conclusion.

3. It is unclear why lines DELD5 and DELA3 are used for Part 1 but only DELB8 is used for Part 2. Does DELB8 show the early phenotypes? It is important to show that the three deletion lines are comparable. Ideally both parts would be completed with all three lines, but if this is not feasible then at least Part 2 should include quantification with an extra control as well as DELD5/DELA3.

4. It is important that graphs are separated per batch in the supplementary figures; however, why do some batches have only deletion organoids? Were deletion organoid experiments not performed alongside controls? RF1 and RF4 in some experiments appear to only have deletion organoids but include controls in other experiments. Because it appears that variability is mainly observed among batches rather than organoids per batch, it is very important that each batch is appropriately controlled (with both control and deletion organoids).

© 2023. Published by The Company of Biologists under the terms of the Creative Commons Attribution License (https://creativecommons.org/licenses/by/4.0/).

Minor changes and clarification:
- The use of “early deletion organoids” and “late deletion organoids” in the abstract is confusing. This wording makes it sound like there is an induction of the deletion either early or late, rather than the analysis of early or late organoids.
- There is a typo on line 263 (“the total cell cycle)
- Genes in line 423 should be in italics
- I appreciate the effort to code the different hiPSC lines in Figure 1D; however, the different control symbols are very difficult to differentiate. Can the authors perhaps have different hues of blue for each line or maybe have crosses like they do for the deletion lines?
- I like the idea of having the same panel from Figure 1A in all figures to guide the reader; however, should it not have a label like the rest of the panels? (eg. Fig 2A)
- It would help the reader to include a dashed line to delineate the edge of the organoids in immunohistochemistry images (and a dashed line around all rosettes as well).
- Please add the DAPI label on Figure 2A
- Please state what each dot in a graph represents in the figure legends or methods: are multiple sections per organoid quantified? How many of them? Is each dot the average of these? (Apologies if this is stated somewhere and failed to find it)
- Are the pictures in Figure 3A from control or deletion organoids? Please state
- Figure 3I is missing the labels
- Why do authors think that the variability observed in deletion organoids is restricted to different batches and not different organoids per batch? What is different in the different batches that is not different in different organoids per batch? The authors briefly mention this in the discussion, but the answer is still not clear.

<u>Reviewer 3</u>

Advance summary and potential significance to field

In this study by Fetit et al, the authors have generated ventral forebrain organoids (spheroids) from isogenic hiPSC lines aimed at modelling deletions at the 16p11.2 locus. The study appears to have been carefully conducted – the experimental design of the study has also carefully thought out. The main findings are centred around increased variability in formation (?) of neural rosettes and expression of a specific ventral telencephalic marker. There are clear impacts on cell cycling and on the type of cells being generated by the deletion hiPSC lines.
Overall, this is an interesting study that explores a novel aspect of 16p11.2 deletion biology.

Comments for the author

I have several comments and suggestions that I’d like to offer in an effort to help improve this study.

- General comment. I was unable to view FigS1. Therefore it was unclear what genes within the 16p11.2 locus were deleted in the isogenic lines – perhaps this could be added elsewhere in the manuscript?
- Was there any evidence of dorsal telencephalic markers in the organoids - particularly in the 16p deletion lines? This may indicate whether or not there is an alteration in the developmental trajectory/cell fate acquisition of these cells (ie cell fated to become interneurons, actually become glutamatergic, for example).
- The assessment of rosette size by assessing the area covered by DAPI positive cells is a good way to examine these structures. However, it is very possible that more subtle phenotypes could be missed. An alternative approach would be to co-stain for of apical membrane (inner lumen), using a maker such as ZO-1 or p-H3 (there are others as well) as well as an early NPC marker (eg SOX2)in oreder to not only assess relative rosette size, but also if rosettes have an intact morphologies and typical arrangement of NPCs around the inner lumen. Of note, previous studies have reported incomplete or altered morphologies of the inner lumen of rosettes generated from hiPSC from autism individuals, which may indicate a common phenotype.
- Are COUPTFII+ cells expected to be only in NPC cells? It is not clear from the example images in fig 2, whether COUPTFII cells were assessed across a whole organoid, or if this was limited to just rosettes. It is also important to consider (If not already done so) that owing to the heterogeneity of

© 2023. Published by The Company of Biologists under the terms of the Creative Commons Attribution License (https://creativecommons.org/licenses/by/4.0/).

organoids that cell density and composition will vary greatly across a organoid. therefore it would be important to assess the number of COUPLTFII cells in multiple (non-consecutive) sections from a single organoid. On top of this, if it were possible to further limit the analysis to another marker (ie early/late NPC or neuronal marker etc) - it would aid in addressing this variability as well as the variability seen in organoid size in the deletion lines.

- It should be noted that TUJ1 is also highly expressed in NPCs as well - this is not a marker of neurons.
- As TUJ1 is expressed in NPCs (late stage NPCs, typically with a fated cell linage, as opposed to SOX2 early stage NPCs) – the flow cytometry analysis does not give an accurate determination of proliferating progenitors and neurons - but rather more about early and late stage NPCs and neurons. To assess for neurons you would ned a specific marker such as NeuN. This should be at least noted in the study, and interpretation of the data modified accordingly.
- Following on, it would be interesting to see what proportion of TUJ1 cells were positive for Ki67.
- fig3I is not labelled, so it is unclear what is being shown here.
- The effect on NeuN expression is very interesting. Is it known what type of cell these NeuN cells are? Eg are they interneurons (SST, calretinin, calbindin etc), or could they be glutamatergic? Please see my comment above about potential differences in cell fate acquisition as a potential phenotype.

First revision

Author response to reviewers' comments

Dear Dr. Guillemot,

Thank you for your email enclosing the reviewers' comments. Please find below a detailed list addressing all the points raised by the reviewers and the corrections undertaken in the manuscript. Briefly, we addressed the major criticisms and have shown that all mutant lines used show the same phenotypes, checked for dorsal identity, investigated timing of neurogenesis, the expression of mature neurons beyond 70 days as well as the expression of interneuron subtypes. Additionally, all minor comments were addressed and edited as per the reviewers' suggestions. All modifications have been highlighted in yellow in the text.

We hope that these revisions merit the manuscript acceptable for publication in Development.

Response to reviewers:

1. Reviewer 1

1.1. Regarding the different lines at the late-stage analysis, the reviewer suggests including control and mutant cell lines from the earlier stages in the latest stage analysis in order to connect the findings.

New data on the two lines used in the late-stage analysis are now added to the early- stage analysis. In **Fig. 1**, immunohistochemical characterisation of the additional lines GM8 and DELB8 (Fig.1B), as well as data on the normalised area and mean organoid size at days 15 and 20 were added (Fig. 1D and E) to connect the findings. The additional data on the lines GM8 and DELB8 agree with the data from the other lines and recapitulate the early-stage phenotypes of the other lines. In **Fig.2 and Fig. S2D and E**, images on the rosette phenotype and COUPTFII expression were also added for the lines GM8 and DELB8, showing that these two lines recapitulate the phenotypes observed in the other lines. **Supplementary Table S3** was edited accordingly to include the raw data for GM8 and DELB8. Data on the lines GM8 and DELB8 were also included in Fig.S2A,B and Fig. S3A and are in agreement with the findings from the other lines.

1.2. The reviewer recommends checking for dorsal identity. Additional IHC staining for the dorsal markers (EMX1 and TBR2) at days 35 and 66, respectively, were added in the supplementary figure (**Fig. S2**) showing no expression of dorsal markers.

© 2023. Published by The Company of Biologists under the terms of the Creative Commons Attribution License (https://creativecommons.org/licenses/by/4.0/).

Regarding the neural identity acquisition, the reviewer raises the concern that there might be issues towards differentiation to neuroepithelium. The protocol used in this study, adapted from Sloan et al., 2018, utilises dual SMAD inhibition to induce rapid neural differentiation of iPSCs and to facilitate neuralization. This is followed by the exposure to BDNF and NT3 to further promote neural maturation. Thus, we expected the differentiation in the ventral organoids to be directed towards neuroepithelial tissue rather than into mesoderm. Nevertheless, we agree that we cannot exclude the possibility of that some non-neuroepithelial cell types are present in our organoids. An analysis of the exact cellular composition of our organoids would require a new round of organoid generation which is beyond the scope of this study. We decided to see if we could find evidence suggesting a systematic genotype-dependent difference in the relative neuroepithelial content of our organoids which, if present, might impact on our interpretations. We quantified the relative mean intensity of the forebrain marker FOXG1 at day 35 using IHC in the cell lines used in parts 1 and 2 of the study (growth and proliferation), as well as the relative mRNA expression of FOXG1 in the cell lines used in part 3 (differentiation) at days 46 and 70 using RT-qPCR and found no significant difference between control and deletion lines in FOXG1 expression **(Fig. S4.** . No significant differences in FOXG1 expression per unit size between the control and deletion organoids suggests no differences in the capacity to differentiate into neuronal tissue in the two genotypes, providing sufficient basis to further investigate the differences in neuronal tissue.

In addition, we added images for the expression of neuroepithelial markers (N-cad and SOX2), showing no observable differences in rosette structures between deletion organoids and control organoids, where rosettes were present, further suggesting that both control and deletion lines appear to differentiate into the neuroepithelium in a similar manner **(Fig.S2**).

1.3. Regarding overall organoid size, the reviewer asks what happens to the organoid sizeat much later stages, beyond 70 days: We agree with the reviewer that it would be interesting to see what happens to organoid size beyond 70 days. Given the time constraint, we are unable to grow and maintain iPSCs for all 7 cell lines, generate organoids and grow them for over 70 days to assess their growth. However, additional data on organoid differentiation for the lines GM8 and DELB8 was added at days 90 and 130 (**Fig.6**) as suggested and addressed in section 1.5.

1.4. The reviewer raises the possibility that mutants are further along in their development relative to absolute time. We agree with the reviewer on the possibility of accelerated development in the deletion lines and have highlighted this, with further elaboration in the additional discussion section (16p11.2 deletion accelerates the developmental trajectory of ventral organoids).

The reviewer also suggests looking into readouts of the neurogenic stage and investigating when does neurogenesis begin. As suggested by Reviewer 3 (and addressed in section 3.5, see below), TUJ1 is expressed both in late NPCs with a determined cell lineage fate and in immature neurons. Therefore, we quantified IHC stains for TUJ1 in the control and mutant lines at day 35, revealing significant increase in the relative mean fluorescent intensity of TUJ1 in the deletion organoids (**Fig 5A**). This was accompanied by a significant increase in the relative mean fluorescent intensity of GAD67 in the deletion organoids at the same time point (**Fig. 5B**). Although this might not tell us when neurogenesis begins exactly, it suggests that the mutant lines are "primed" towards neurogenesis at this stage of the analysis and are further along in their developmental stage, compared to controls.

1.5. The reviewer suggests that precocious development would be expected to lead to fewer interneurons at the end and suggests examining the organoids at later stages. Late stage examination of the lines GM8 and DELB8 was performed as suggested by the reviewer (**Fig. 6, Table S7**). We quantified the relative mean fluorescent intensity of NEUN and LHX6 for the lines DELB8 and GM8 at days 50, 90 and 130. Indeed, a significant increase in both NEUN and LHX6 was observed in the deletion organoids at day 50, followed by a significant reduction in LHX6 at day 130 (**Fig. 6C-E**). This is in line with the reviewers' interpretation of the mutant lines, where the accelerated development in the deletion organoids would result in fewer interneurons at later stages. This is also further highlighted in the discussion.

1.6. All minor comments have been addressed:

1.6.1. The title has been changed as per the reviewer's suggestion to convey the findings more

© 2023. Published by The Company of Biologists under the terms of the Creative Commons Attribution License (https://creativecommons.org/licenses/by/4.0/).

clearly.

1.6.2. A “Replicate” within a batch accounts for the organoids grown from a distinct, separate iPSC colony. The explanation has been added to the study design in **Supplementary Table S1**.

2. Reviewer 2

2.1. The reviewer recommends including additional data on the fate and specification of the ventral organoids and immunohistochemistry quantification for ventral forebrain markers at the early time point, as well as markers for other CNS regions.

The forebrain marker FOXG1 and the ventral forebrain markers (GSX2 and NKX2.1) were quantified at day 35 by examining their mean intensity relative to organoid size from IHC staining (**Fig. S4A-D, Table S10**). Additional staining to check for dorsal identity was included (**Fig. S2A-D**), as requested by reviewer 1 as well (section 1.2 above).

2.2. The reviewer suggests quantifying additional interneuron markers in addition to NeuN: The mean fluorescent intensity of the GABAergic marker GAD67 was quantified relative to organoid size at day 35 **(Fig. 5B and D, Table S11).** LHX6 quantification was performed at days 50, 90 and 130 **(Fig. 6A-E, Table S7)**. Additional RT-qPCR data investigating the expression of interneuron markers was also included (**Fig.6F and G**).

2.3. The reviewer asks whether the deletion line DELB8 used in the later parts of the study show the early phenotypes. Indeed, the line DELB8 recapitulates the early phenotypes observed in the lines DELD5 and DELD3. We included additional data for the lines in (DELB8 and GM8) in **Fig.1** and **Fig.2** showing that the three deletion lines are comparable. This also addresses Reviewer 1’s comment (section 1.1 above)

2.4. The reviewer asks why some batches have only deletion organoids without control organoids. All batches initially included at least one control and one deletion line for comparison. However, some control samples in the batch RF4 were lost during processing and sectioning. Similarly, some organoids were excluded from the flow cytometric analysis on the basis that too few SOX2+Ki67+ cells remained after doublet exclusion to perform rigorous cell cycle analysis. This has been clearly stated and addressed in detail in the legends of **Fig.S3** and **Fig.S5**. Moreover, we used Linear Mixed Effects (LME) models in our statistical analysis, accounting for the variability due to the different batches and different cell lines. We agree with the reviewer that it is not ideal not having controls for some batches, however, our statistical approach accommodates for that.

2.5. All minor comments have been addressed:

2.5.1. “early deletion organoids” and “late deletion organoids” have been changed to “At early stages…” and “At later stages…” as per the reviewer’s suggestion.

2.5.2. the typo was corrected

2.5.3. Genes were italicised

2.5.4. Different colour hues and point shapes were used for the different genotypes and lines in Fig.1D.

2.5.5. The same guiding panel has been used in all figures.

2.5.6. Dashed lines to delineate the organoid edge and rosettes have been added to Fig. 2A and B.

2.5.7. DAPI has been labelled on Fig.2A

2.5.8. We stated what each dot represents in each figure legends and in the materials and methods.

2.5.9. We stated whether the representative images in Fig 3A were from deletion or control organoids in the figure legends. We used a representative image from the deletion organoid simply because rosettes were very small and scarce in the controls.

2.5.10. Fig.3I has been labelled

2.5.11. We believe that the variability observed in the deletion organoids is primarily due to the different batches and not the different organoids per batch. This is owing to the materials and reagents used in generating the batches, such as Fetal Bovine Serum, KnockOut Serum Replacement, and B27 supplements which are susceptible to lot-to-lot variability. Whilst those are the same reagents from the same supplier, their constitution might slightly vary at the time of purchase. This is also highlighted in the initial protocol by Sloan et al., 2018.

© 2023. Published by The Company of Biologists under the terms of the Creative Commons Attribution License (https://creativecommons.org/licenses/by/4.0/).

3. Reviewer 3

3.1. The reviewer was unable to view Fig. S1 and the genes within the 16p11.2 locus: The figure has been edited to ensure it is viewable and the genes deleted within the locus have been clearly highlighted in **Supplementary Table S2**.

3.2. The reviewer asks whether there was any evidence of dorsal telencephalic markers in the organoids, particularly in the 16p deletion lines. Additional markers for dorsal identity were included (**Fig. S2A**), as per the suggestions of all 3 reviewers (also addressed in sections 1.2 and 2.1 above). No evidence of dorsal telencephalic identity was found.

3.3. The reviewer recommends using an alternative approach to co-stain for apical membrane (inner lumen) markers and early NPC marker to assess rosette morphology.
Additional staining for SOX2 and N-cadherin were added to characterise rosettes (**Fig.S2B**). No differences in either rosette morphology or NPC arrangement around the inner lumen was observed.

3.4. The reviewer asks whether COUPTFII cells were assessed across a whole organoid, or if this was limited to just rosettes and highlights the importance of considering the variability in cell density across the individual organoids. Because COUPTFII expression is not limited to rosettes, its expression was quantified in the whole organoids and not just in the rosettes, especially since the control organoids rarely formed any rosettes at all. This was stated in the figure legend (**Fig. 2B**).

We agree with the reviewer that organoids are heterogenous, and that cell density and composition could vary across an organoid. Thus, we consistently assessed the expression of COUPTFII in sections from the middle of the organoid. This was stated and highlighted in the manuscript.

3.5. The reviewer highlights that TUJ1 is also highly expressed in NPCs, specifically the late-stage NPCs with a fated cell linage, unlike SOX2 which marks early-stage NPCs and recommends examining the proportion of TUJ1+Ki67+ cells in the flow cytometric analysis. The interpretation of the data was corrected to state that TUJ1 is a marker of late stage NPCs as per the reviewer's suggestion. The proportion of cycling late NPCs (TUJ1+Ki67+/SOX2+) was also quantified as well as the ratio of late:early (TUJ1+Ki67+/SOX2+Ki67+) NPCs (**Fig.S5C-F and Table S9**) as per the reviewer's suggestion.

3.6. The reviewer asks to label Fig. 3I. The figurehas been labelled.

3.7. The reviewer asks about the type of NEUN+ cells to highlight differences in cell fate acquisition as a potential phenotype. Additional RT-qPCR analysis was performed to investigate the expression of the interneuron subtypes: SST, RELN, CB, CR and NPY (**Fig. 6F,G** and **Fig. S7A,B**).

Second decision letter

MS ID#: DEVELOP/2022/201227

MS TITLE: 16p11.2 deletion accelerates subpallial maturation and increases variability in human iPSC-derived ventral telencephalic organoids

AUTHORS: Rana Fetit, Michela Ilaria Barbato, Thomas Theil, Thomas Pratt, and David J. Price

I have now received the reports of the three referees who reviewed the earlier version of your manuscript and have reached a decision. The referees' comments are appended below, or you can access them online: please go to BenchPress and click on the 'Manuscripts with Decisions' queue in the Author Area.

The reviewers' overall evaluation is positive and we would like to publish a revised manuscript in Development, provided that you satisfactorily address the remaining minor suggestions and

© 2023. Published by The Company of Biologists under the terms of the Creative Commons Attribution License (https://creativecommons.org/licenses/by/4.0/).

comments of referees 1 and 2. Please attend to all of the reviewers' comments in your revised manuscript and detail them in your point-by-point response. If you do not agree with any of their criticisms or suggestions explain clearly why this is so.

Reviewer 1

Advance summary and potential significance to field

The revised manuscript by Rana et al. has been improved with several new stainings and quantifications, and the mechanism and final effect has been more clearly discerned. Thus, I feel this manuscript is now acceptable for publication.

Comments for the author

I have only two minor comments that would be good to address before publication:

1. The inclusion of data from GM8 and DELB8 lines in the earlier characterisation is good, but why are these labelled “Differentiation” in Figure 1? My understanding is that “Differentiation” refers to the later time points of days 46-130, but 1B and C are analyses performed at day 33-35 and day 25, so if these lines were also analysed at these earlier time points I suggest simply incorporating them in the mutant and control groups. If these stainings and images were not taken at these same earlier time points, however, then this should be made clear and ideally data at these early time points included, as initially suggested. In addition, contrary to what is stated in the response letter, GM8 and DELB8 do not seem to be included in Figure 2.

2. The staining for NeuN and LHX6 at very late stages is a very good addition, and strongly supports the interpretation of precocious differentiation. The lack of difference in NeuN staining at the latest stage tested is somewhat unexpected, as I would have thought it would be decreased by these late stages. Given that LHX6 is involved in interneuron migration and therefore expressed in more immature neurons, while NeuN is expressed in more mature neurons, perhaps the lack of decreased NeuN is simply due to not having analysed late enough. A later staining is not necessary, but this could at least be mentioned in the discussion as a future direction.

Reviewer 2

Advance summary and potential significance to field

The manuscript by Fetit et al use ventral forebrain organoids derived from induced pluripotent stem cells to investigate the effect of the 16p11.2 deletion in ventral progenitor proliferation, differentiation and fate. 16p11.2 is associated with ASD and other neurocognitive phenotypes at varying degrees of penetrance, and while a number of studies have investigated the role of genes within the 16p11.2 region in developmental processes related to excitatory cortical neurons, no study has, of yet, studied their role in interneuron development.

Comments for the author

The authors addressed most of the points brought up in the first revision. In this revision there are a few more things that I believe need clarification. Please see below:

- While I appreciate the effort to validate the early time point phenotypes in the two lines used in the second part of the study, I don’t think this point is completely addressed. The authors state “This was consistent in all 3 deletion lines used in the different parts of the study” when looking at rosette size in Figure 2. However, the data for the third line (DELB8) includes only an immunohistochemistry panel with no quantification and includes organoids at Differentiation stages (rather than the Growth and Proliferation stage as for the other deletion lines). At a minimum, the authors should re-phrase the sentence underlined above so as to reflect the data more accurately (a similar thing can be said for the statement about increased COUPTFII expression in DELB8)

© 2023. Published by The Company of Biologists under the terms of the Creative Commons Attribution License (https://creativecommons.org/licenses/by/4.0/).

- It appears as if NeuN and LHX6 expression decreases over time in both control and deletion organoids in Figure 6C. Are neurons dying over time?

- In addition, data in Figures 6B and 6E show a decrease of LHX6 expression and relative mean intensity in deletion organoids. The authors state “The significant reduction of LHX6 at days 70 and 130 further supports the notion that this deletion causes premature differentiation into interneurons”. However, it is my understanding that LHX6 is retained in postmitotic interneurons, so precocious birth of interneurons would lead to increased gabaergic markers at progenitor stages (as observed), increased neuronal markers early in the differentiation stages (as observed), but a plateau of LHX6 over time where the controls catch up to the deletion pace. Please explain.

Other minor comments:
- In highlighted section of second paragraph in page 8, please state if this refers to control or deletion organoids
- Similarly, please state that Figures 3A-D represent deletion organoids (in the figure as well as figure legend)
- Figure 3C could benefit from increasing the intensity of the green channel (it is barely visible right now)
- There is a typo on line 275: should read “exhibited increased total cell cycle lengths”
- There is a typo on line 292: should read “used to calculate the total”
- Add “in deletion organoids” in lines 363-365
- There is a typo on line 382: should read “MGE-derived”
- Add a DAPI label in Figure 6C

Reviewer 3

Advance summary and potential significance to field

In this study by Fetit et al, the authors have generated ventral forebrain organoids (spheroids) from isogenic hiPSC lines aimed at modelling deletions at the 16p11.2 locus. The study appears to have been carefully conducted – the experimental design of the study has also carefully thought out. The main findings are centred around increased variability in formation (?) of neural rosettes and expression of a specific ventral telencephalic marker. There are clear impacts on cell cycling and on the type of cells being generated by the deletion hiPSC lines. Overall, this is an interesting study that explores a novel aspect of 16p11.2 deletion biology.

Comments for the author

The authors have addressed all of my concerns.

Second revision

Author response to reviewers' comments

Dear Dr. Guillemot,

Thank you for your email enclosing the reviewers’ comments. We are very pleased that the reviewers find our manuscript acceptable for publication, albeit with a few minor revisions which we have addressed. Please find below a detailed list highlighting all the points raised by the reviewers and the corrections undertaken in the manuscript. Briefly, we stated clearly that images of organoids from the different lines were analysed at the same time points and removed any misleading labelling that implied otherwise. In addition, we added further elaboration on the NEUN and LHX6 findings in the discussion for a better interpretation of the results. All modifications have been highlighted in yellow in the text.

© 2023. Published by The Company of Biologists under the terms of the Creative Commons Attribution License (https://creativecommons.org/licenses/by/4.0/).

Reviewer 1

1. The reviewer suggests incorporating the lines DELB8 and GM8 in their corresponding mutant and control groups in Fig. 1, and asks why are the lines GM8 and DELB8 not included in Fig. 2 as stated in the response letter.

We have added the lines GM8 and DELB8 to their respective control and deletion groups in Fig. 1B and C and removed the labels “differentiation” to avoid confusion.

The data for the lines GM8 and DELB8 have been moved to supplementary Fig. S2, since they were not quantified in Fig. 2. We apologise for this typo in the initial response letter. Please find the data on lines GM8 and DELB8 in Fig. S2D and E.

2. The reviewer states that LHX6 is involved in interneuron migration and is, therefore, expressed in more immature neurons, while NEUN is expressed in more mature neurons and requires further elaboration on the lack of difference in NEUN staining at 130 days.

We agree with the reviewer’s interpretation that this might be owing to the maturity of interneurons. We have elaborated on that in the discussion, in the section “16p11.2 deletion accelerates the developmental trajectory of ventral organoids”, Lines 520-523.

Reviewer 2

1. The reviewer states that the data for the line (DELB8) includes only an immunohistochemistry panel with no quantification and includes organoids at Differentiation stages (rather than the Growth and Proliferation stage as for the other deletion lines. The reviewer also recommends to re-phrase the text to reflect the data more accurately for rosettes and COUPTFII expression.

The label “Differentiation” was initially used to refer to the lines used to investigate the differentiation aspect of organoids (part 3) and not the stage at which these panels were taken. We would like to bring to the reviewer’s attention that all the Immunohistochemistry images included are indeed at the same stage as the other deletion lines (days 33-35). This point was also raised by reviewer 1 (point #1.1). Therefore, to avoid confusion, we have removed the labelling “Growth and Proliferation” and “Differentiation” in Figures 1 and 2 as well as Figure S2. We have also added the days at which the images were taken (Fig. S2D and E). As per the reviewer’s suggestion, we have also rephrased the sentence highlighted to state that all the images were taken at the same timepoints and that the data on DELB8 and GM8 were not quantified (Lines 189-191 and 220-22).

2. The reviewer asks whether the reason NeuN and LHX6 expression decreases over time in both control and deletion organoids in Figure 6C is due to neurons dying over time.

We have performed a preliminary T-test to confirm the reduction in NEUN and LHX6 expression between days 90 and 130 in both control and deletion organoids (Supplementary table S7). The reductions were significant, and we agree with the reviewer’s interpretation that interneurons might be dying. We speculate that the lack of excitatory cortical neurons means no integration into function circuits which might contribute to interneuron death. Another technical reason could be that when organoids are maintained for a long time (130 days or more), their core becomes necrotic due to the lack of diffusion of nutrients and gases, which might contribute to the proposed neuronal death.
However, performing viability or TUNNEL assays at 130 days to specifically confirm neuronal death was beyond the scope of our study.

3. The reviewer states that: precocious birth of interneurons would lead to increased GABAergic markers at progenitor stages (as observed), increased neuronal markers early in the differentiation stages (as observed), but a plateau of LHX6 over time where the controls catch up to the deletion pace, and requires an explanation.

© 2023. Published by The Company of Biologists under the terms of the Creative Commons Attribution License (https://creativecommons.org/licenses/by/4.0/).

We agree with the reviewer that if precocious development was the only factor at play, then we'd expect a plateau of LHX6 at later time points. However, this locus contains over 20 protein coding genes which converge on multiple pathways. Our work demonstrates only one effect of the deletion, namely: precocious development. However, we cannot state with certainty that this is the only outcome, and other factors may come to play owing to the loss of this locus, such as neuronal survival and death (as suggested by reviewer 2, point #3) and neuronal migration (as suggested by reviewer 2, point #2). Thus, we have highlighted that the reduction in LHX6 is unexpected and that other factors that require further investigation might play a role (Discussion, Lines 525-528).

minor comments:

1. **In highlighted section of second paragraph in page 8, please state if this refers to control or deletion organoids.**
   This has been edited accordingly (Lines 241-242).

2. **Similarly, please state that Figures 3A-D represent deletion organoids (in the figure as well as figure legend).**
   This has been added to the Figure 3 and legend (Line1016)

3. **Figure 3C could benefit from increasing the intensity of the green channel**
   The intensity of the green channel has been increased so that it is more visible.

4. **There is a typo on line 275: should read "exhibited increased total cell cycle lengths"**
   The typo has been corrected (Line 270)

5. **There is a typo on line 292: should read "used to calculate the total"**
   This has been corrected (Line 287)

6. **Add "in deletion organoids" in lines 363-365**
   This has been added (Line 358-359)

7. **There is a typo on line 382: should read "MGE-derived"**
   This has been corrected (Line 376)

8. **Add a DAPI label in Figure 6C**
   DAPI label has been added to the figure.

Third decision letter

MS ID#: DEVELOP/2022/201227

MS TITLE: 16p11.2 deletion accelerates subpallial maturation and increases variability in human iPSC-derived ventral telencephalic organoids

AUTHORS: Rana Fetit, Michela Ilaria Barbato, Thomas Theil, Thomas Pratt, and David J. Price
ARTICLE TYPE: Research Article

I am delighted to tell you that your manuscript has been accepted for publication in Development, pending our standard ethics checks.

© 2023. Published by The Company of Biologists under the terms of the Creative Commons Attribution License (https://creativecommons.org/licenses/by/4.0/).
